# Spirometry Reference Equations Including Existing and Novel Parameters

**DOI:** 10.2174/18743064-v16-e221227-2022-14

**Published:** 2023-02-23

**Authors:** Douglas Clark Johnson, Bradford Gardner Johnson

**Affiliations:** 1 Department of Medicine, Baystate Medical Center 759 Chestnut Street Springfield, MA 01199, USA; 2 Washington, D.C. 20010 USA

**Keywords:** Forced vital capacity, Interstitial lung disease, Peak expiratory flow, Pulmonary function tests, Reference equations, Spirometry

## Abstract

**Introduction:**

Spirometry is an essential component of pulmonary function testing, with interpretation dependent upon comparing results to normal. Reference equations for mean and lower limit of normal (LLN) are available for usual parameters, including forced vital capacity (FVC), forced expiratory volume in the first second of an FVC maneuver (FEV1), and FEV1/FVC. However, standard parameters do not fully characterize the flow-volume loop and equations are unavailable for the upper limit of normal (ULN). The aim of this study was to develop reference equations for existing and novel spirometry parameters, which more fully describe the flow-volume loop, and to compare these to previously reported equations.

**Methods:**

Data from healthy participants in NHANES III was used to derive reference equations for existing and novel spirometry parameters accounting for birth sex, age, height, and ethnicity (Caucasian, Mexican American, Black) for ages 8 to 90 years. An iterative process determined %predicted LLN and ULN. Equations were compared to published reported equations.

**Results:**

Reference equations were developed for mean, LLN and ULN for existing and novel spirometry parameters for ages 8 to 90. The derived equations closely match mean values of previously published equations, but more closely fit the LLN. Mexican-American and Caucasian values were similar (within 2%) so they were combined, while Black relative to Caucasian/Mexican-American values were lower for some parameters.

**Conclusion:**

These reference equations, which account for birth sex, age, height, and ethnicity for existing and novel spirometry parameters, provide a more comprehensive and quantitative evaluation of spirometry and the flow-volume curve.

## 1 INTRODUCTION

Spirometry is the mainstay of pulmonary function testing (PFT). Interpretation of spirometry is critically dependent upon comparing results to normative values for healthy individuals of the same birth sex, age, height, and ethnicity calculated from reference equations. A result is generally considered abnormal when below 5^th^ percentile or lower limit of normal (LLN). However, for some parameters, diseases lead to results above the 95^th^ percentile or upper limit of normal (ULN).

The 2017 American Thoracic Society (ATS) guidelines for PFT reporting [1] recommend “Only FVC, FEV1, and FEV1/FVC need be routinely reported”. However, the 2019 ATS update on spirometry [2] notes “Although FEV1 and FVC are the primary parameters measured in spirometry, there is far more information contained in the flow and volume data. Continuing research on innovative analyses that may improve diagnoses or lead to earlier diagnosis in at-risk persons is important ….”.

The shape of the flow-volume curve during an expiration made as forcefully and completely as possible starting from full inspiration can be represented by different parameters that can provide more information about lung function including elastic recoil which is increased in interstitial lung disease or of upper airway obstruction. Spirometry parameters include FEF25–75%, forced expiratory flow at 25–75% of FVC; FEF75%, the flow at 75% of FVC; FEV05, FEV1, FEV3, FEV6 the expiratory volume in the first 0.5, 1, 3, or 6 seconds of an FVC maneuver; FVC, forced vital capacity; and PEF, peak expiratory flow. There are currently no predictive equations for PEF/FVC or FEF25-75%/FVC, which are often elevated in patients with interstitial lung disease, nor for PEF/FEV1, which is often reduced in patients with upper airway obstruction [3]. Establishing normative prediction equations for these and other parameters could allow improved and earlier diagnosis.

The National Health and Nutrition Examination Survey (NHANES) III study includes spirometry measurements and has been used to determine prediction equations for several spirometry values for “normal” subjects for ages 8 to 80 [4]. Since NHANES III collected spirometry on subjects to age 90, prediction equations up to age 90 can be determined. While the Global Lung Initiative (GLI) [5] reports mean and LLN multiethnic equations for FEV1, FVC, FEV1/FVC, FEF25-75% and FEF75% for age 3-90 or 3-95, it does not provide equations for FEV3, FEV6, PEF, for ratios other than FEV1/FVC or provide ULN equations.

This study uses NHANES III data to determine normative reference equations for existing, additional, and novel parameters for Caucasian/Mexican-American and Black ethnic groups and provides equations for % predicted LLN and ULN using an iterative method. Having these normative values for additional parameters will improve clinicians’ ability to interpret spirometry on a quantitative basis and allow research into the clinical value of the additional and novel parameters.

## 2 MATERIALS AND METHODS

### 2.1 Study Design and Creation of Spirometry Parameters and Regression Equations

The third National Health and Nutrition Examination Survey (NHANES III) was conducted from 1988 to 1994 of a random sample of the U.S. population living in households. The adult file includes subjects 17 and over, and youth file includes subjects aged 8-17. Spirometry measurement from NHANES III has been described [4], with spirometry values including FEF75%, FEV05, FEV1, FEV3, FEV6, FVC, FEF25-75%, PEF, and FEV1/FVC. The method of importing and merging NHANES III data sets [6] included merging the adult and youth files matching on SEQN field. Then parameters FEV1/FEV3, FEV1/FEV6, FEV1/FVC, FEV3/FVC, FEV6/FVC, and the novel parameters FEF75%/FVC, FEF75%/PEF, FEV05/FEV3, FEV05/FVC, FEV3/FEV6, FEF25-75%/PEF, FEF25-75%/FVC, PEF/FEV1, PEF/FEV6, and PEF/FVC were created. Novel and other parameters include FEF25-75%/FVC, FEF25-75%/PEF, FEF75%/FVC, FEF75%/PEF, FEV05/FEV3, FEV05/FVC, FEV1/FEV3, FEV1/FEV6, FEV3/FEV6, FEV3/FVC, FEV6/FVC, PEF/FEV1, PEF/FEV6, and PEF/FVC. While most ratios are expressed as percentage, ratios of flow/volume are expressed as fraction.

Subsets of “normal” adult and youth subjects were first determined using age and exclusion criteria similar to Hankinson as described in the Appendix (Tables **1** and **2**). Additional subsets based on ethnicity, age, and birth sex were determined from the “normal” adult and youth subjects taken from the youth file combined with those from the adult file meeting age criteria. Ethnicity groups included Caucasian, Black, Mexican-American, and Caucasian/Mexican-American. Age/birth sex groups included males 20 and over, females 18 and over, males under 20, and females under 18.

Prediction equations for each subset were determined for every spirometry parameter. Linear regression was done by a method identical to that of Hankinson [4]. These equations for each subset were then used to create predicted values and %predicted values for each parameter for every subject.

### 2.2 Comparison to other Studies

It should be noted that Hankinson used a data set not released to the public (personal communication from J.L. Hankinson), so small differences were expected. To compare the prediction equations of this study to those of Hankinson [4], the mean and standard deviation of the difference between the %predicted values of this study and those of Hankinson were determined for each parameter and ethnicity. Since there was little difference between the 8-80 and the 8-90 groups, only comparisons of the 8-90 subjects are reported.

To assess the prediction equations for a lower limit of normal of Hankinson [4] and Hansen [7], the fraction of subjects with values below those LLN was then determined for each subset, and the fraction of patients below the LLN by age was also determined.

### 2.3 Effects of Race-ethnicity and Age

To determine the effect of race-ethnicity on spirometry, %predicted of Caucasian and %predicted of Caucasian/Mexican-American were determined for each subject, then the mean and standard deviation for each subset was calculated. Parameters best %predicted value were determined using Caucasian/Mexican-American prediction equations for Caucasians and Mexican-Americans and the Black prediction equations for Blacks. Then subsets of all subjects of each ethnicity were determined. Combining Black, Caucasian, and Mexican-American created a set of all subjects.

The mean (which should be 100%) and standard deviation of the best %predicted value for each parameter and subset were determined. Scatter plots of the best %predicted *versus* age for each parameter were viewed to evaluate how the distribution varied by age.

### 2.4 Determination of LLN and ULN

An iterative method was used to determine limits so exactly 5% of subjects were below the LLN and 5% above the ULN of the best %predicted for each parameter. This found that fewer subjects age<50 were below the LLN or above the ULN compared to those ≥50, indicating that a single LLN or ULN of %predicted could not be used for all ages. The final method used the iterative method to determine the LLN and ULN of %predicted for subjects <50 and for subjects ≥50. Then the fraction of subjects below the LLN or above the ULN for each parameter was determined in 2-year intervals using a rolling 4-year interval.

### 2.5 Detailed Method to Determine LLN and ULN

The fraction of subjects with values below the lower limit of normal (LLN) or above the upper limit of normal (ULN) under the assumption that the parameters were normally distributed (= mean * (1 ± SD)) was determined. An iterative method was then used to determine limits so exactly 5% of subjects would be below the LLN and 5% above the ULN of the best %predicted for each parameter. The best % predicted used Caucasian/Mexican-American equations for Caucasians and Mexican-Americans and Black equations for Blacks. The initial limits were set to 100 ± SD * 1.645. For each iteration if there were more than 5% below the LLN or fewer than 5% above the ULN the limit was decreased by a difference (which started at 2). When fewer than 5% were below LLN or more than 5% above the ULN, the limit was increased by the difference. The difference was halved each time there was an overshoot, with a total of 30 iterations. Then the fraction of subjects below the LLN or above the ULN for each parameter was determined in 2-year intervals from age 8 to 90 for those within 24 months of each age (i.e rolling 4-year groups except 2-year groups for ages 8-10 and 88-90). This found that fewer subjects below age 50 were below the LLN or above the ULN compared to those 50 and over, indicating that a single LLN or ULN of %predicted could not be used for all ages. The final method to determine LLN and ULN was to use the iterative method described above to determine the LLN and ULN of %predicted for subjects under age 50 and for subjects 50 and older.

### 2.6 Statistical Analysis

Linear regression was performed using LinearRegression from sklearnlinear in sklearn [8] model in the form b0+b1*age for ratio parameters and in form b0+b1*age+b2*age^2^ +b3*height^2^ for non-ratio parameters. Age in years was calculated from age in months at the time of the exam/12 and height in centimeters. Histograms and scatterplots of spirometry parameters were created using matplotlib.

Patient and Public Involvement: There was no patient or public involvement in the development or design of this study which evaluated NHANES III data sets.

## 3 RESULTS

Of the 16840 adults and 4146 youth who performed spirometry, 4863(29%) adults aged 17- 80, 5072(30%) adults aged 17-90 (Appendix Table **1**), and 2779(52%) youth aged 8 to 17 (Appendix Table **2**) remained to be analyzed after applying the exclusion criteria. There were 7851 subjects aged 8-90 including 2464 Caucasian, 2623 Black, and 2764 Mexican-American with age distribution shown in Fig. (**1**). There were minor differences in the numbers of subjects from those of Hankinson analysis of NHANES III [4], which included 4634 adults under age 80 and 2795 youth.

Prediction equations derived from linear regressions from subjects 8-90 are shown in Table **1a** (male) and Table **1b** (female) for non-ratio parameters and in Table **2a** (male) and Table **2b** (female) for ratio parameters.

Regression equations from this study were very close to those of Hankinson (Appendix Table **3**). The difference was <1% for all parameters, with a standard deviation <2% for all parameters except for FEF25-75% and PEF. When we compared regressions based on males 20-80 to 20-90 and females 18-80 to 18-90, there was little difference up to age 65, but differences at higher ages with generally higher predicted values among subjects over age 65 from equations derived from the up to 90 group (results not shown).

Mexican-American and Caucasian %predicted values were very similar, within 2% for all parameters (results not shown), while Blacks were different, validating combining Mexican-American and Caucasian groups for the best regression. Blacks had 15% lower FEV1, 16% lower FVC, 11% lower FEF25-75%, identical FEV1/FVC, 5% lower PEF, and 11% higher PEF/FVC as Caucasian/Mexican-American (Appendix Table **4**).

Using Hankinson equations for LLN, most parameters had subgroups with <4% or > 6% of subjects below the LLN, ranging from 2.2% to 8.0% (Appendix Table **5a**). Using Hansen’s equations, adult subgroups had from 3.4% to 6.3% of subjects below the LLN (Appendix Table **5b**).

**Fig. (2):**
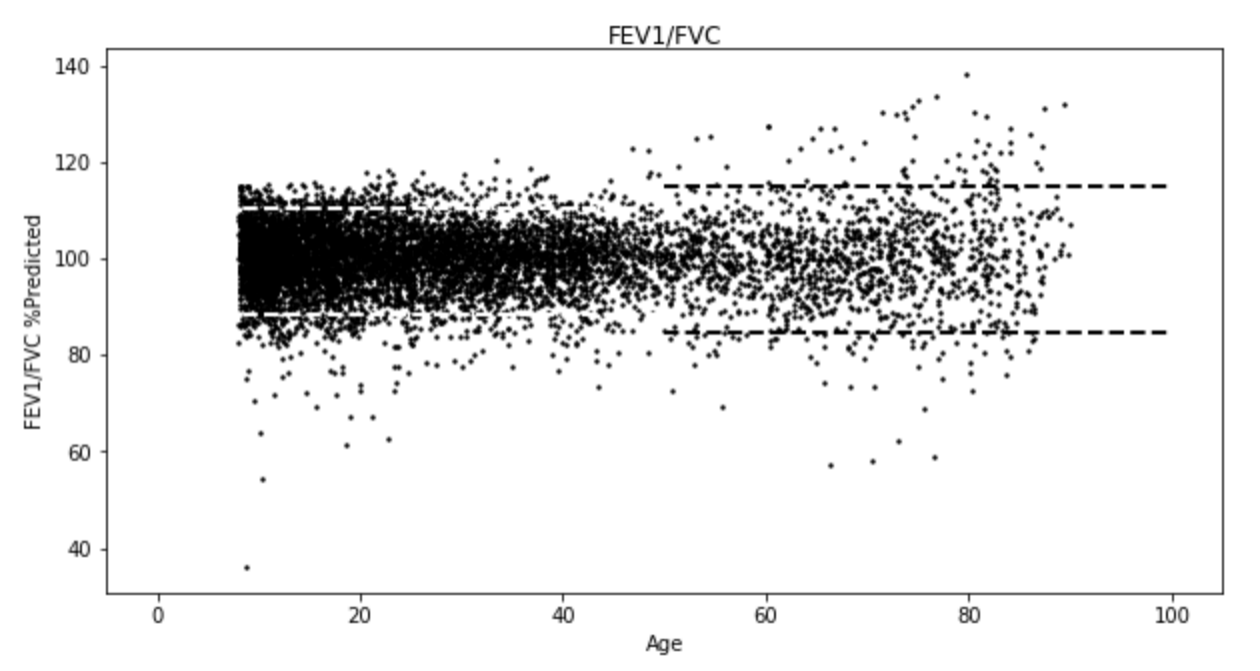
Scatterplot of FEV1/FVC percent of best predicted values by age with LLN (88.5, 84.7) and ULN (110.5, 114.9) for age <50 and for age ≥50. (e-Figure 1 has scatterplots of all parameters.)

Scatter plots of best % predicted *versus* age showed a wider variation for those ≥50 compared to those <50 (Fig. **2**) for FEV1/FVC and Appendix Fig. (**1**) for all parameters). Assuming a normal distribution of %predicted values, many parameters had more or fewer than 5% of subjects below the LLN or above the ULN (results not shown), indicating that many parameters were not normally distributed. The iterative method determined lower and upper limits, so exactly 5% of all subjects below the LLN or above the ULN for each parameter %predicted, which with standard deviation allowed calculation of z-scores. Table 3 shows %predicted LLN, ULN, and standard deviation for each parameter for all subjects and for those <50 and those ≥50, along with z-scores for the LLN (LLNz=(100-LLN)/SD) and ULN (ULNz=(ULN-100)/SD) for all subjects. Since we determined the LLN and ULN from the exact numbers of subjects in the lower or upper 5%, we did need to adjust a normal distribution for skewness or kurtosis. Many parameters demonstrated skewness with LLNz differing from ULNz. The LLN and ULN for those <50 were near 80% and 120% for some parameters, including FEV1, FEV3, FEV6, and FVC but other parameters, including FEF25-75%, FEF75%, and PEF had a wider distribution. The LLN and ULN for ratios had a wider distribution for FEF25-75%/PEF and PEF/FVC than for FEV1/FVC.

**Fig. (3):**
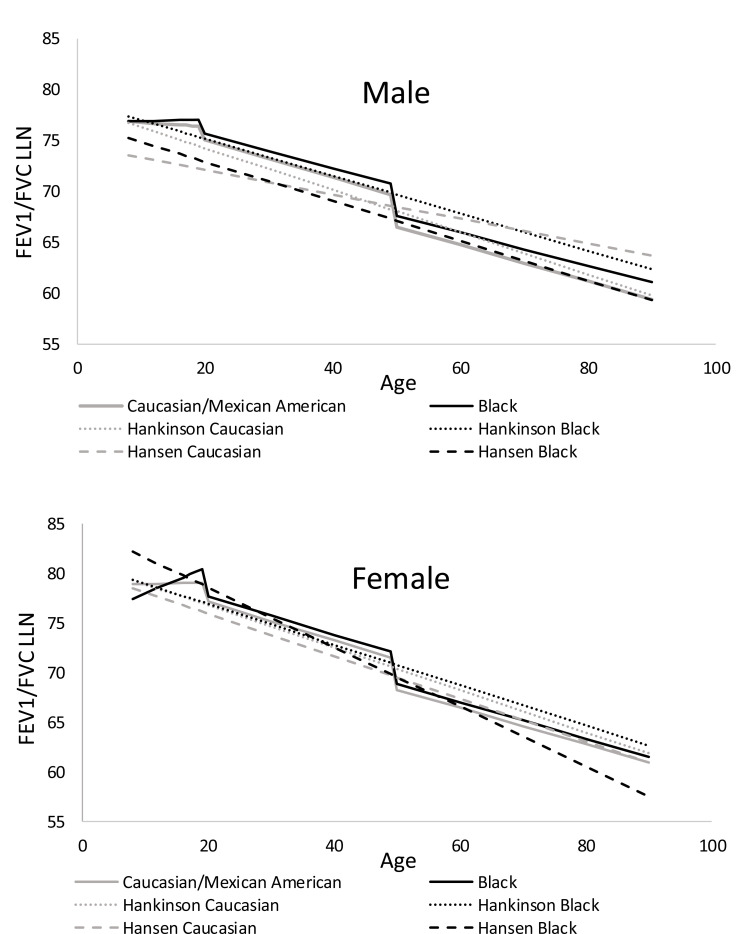
FEV1/FVC Lower Limit of Normal (expressed as percentage) by age for Caucasian/Mexican-American and Black from this study, Caucasian and Black (Hankinson), and Caucasian and Black (Hansen) for male and female.

**Fig. (4):**
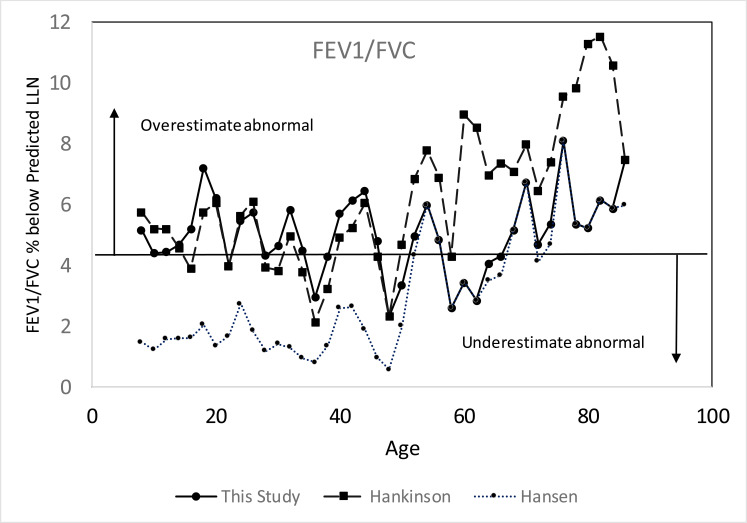
Percentage of subjects with FEV1/FVC below predicted lower limit of normal (LLN) by age (those ± 2 years, overlapping 4-year intervals) from this study, Hankinson, and Hansen.

**Fig. (5):**
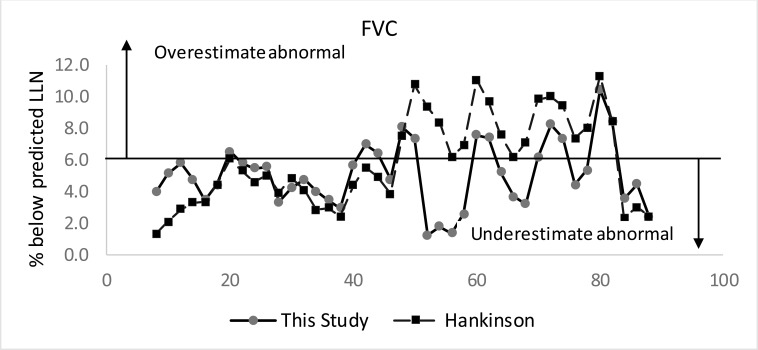
Percentage of patients with FVC below the predicted LLN by age (those ± 2 years, overlapping 4-year intervals) from this study and Hankinson.

There are differences in predicted LLN for FEV1/FVC from this study and those of Hankinson and Hansen (Fig. **3**), including less difference by ethnicity from this study. The percentage of subjects having FEV1/FVC below the LLN by age from this study differed from those of Hankinson and Hansen (Fig. **4**), and the percentage of subjects having FVC below the LLN by age differed from those of Hankinson (Fig. **5**).

Fig. (**6**) shows the percentage of subjects having FEV1/FVC below the LLN or above the ULN by age, with Figure e2 showing percentage below the LLN or above the ULN for all parameters.

Fig. (**7**) shows pulmonary function test from a patient with interstitial lung disease having normal FEV1, FVC and unadjusted KCO but elevated FEF25-75%/FVC and PEF/FVC and reduced TLC and DLCO adjusted for lung volume.

## 4 DISCUSSION

Interpretation of spirometry depends upon knowing normative values based on patient characteristics. Accurate interpretation of spirometry also depends upon an assessment of the shape of the flow-volume curve, not simply the %-predicted values of traditional parameters such as FEV1, FVC, FEV1/FVC, and PEF. This study provides normal, lower and upper limits of normal for existing and novel spirometry parameters using data from NHANES III, providing a more comprehensive quantitative assessment of the normal range of spirometry. For non-ratio parameters much of their variation could be accounted for using regressions including age, age^2^, and height^2^ with parameters decreasing with age and increasing with height. Ratio parameter showed little change in youth. While most ratio parameter decreased with age in adults, some had no change and PEF/FEV1 increased with age.

The shape of the flow-volume loop has the potential to provide important information about underlying physiology [9]. Interstitial lung disease can increase lung elastic recoil leading to increased PEF relative to FVC and a convex shape with increased FEF25-75% and FEF75% relative to PEF - even when FEV1 and FVC are within normal limits. On the other hand, the obstructive disease leads to a concave expiratory curve with reduced FEF25-75% and FEF75% relative to PEF, even when FEV1 and FVC are within normal limits. If FEV1 and PEF are reduced due to severe respiratory muscle weakness, a concave curve is not expected, so FEF25-75% and FEF75% would not be reduced relative to PEF. With fixed or intrathoracic upper airway obstruction, there is a plateau of expiratory flows with reduced PEF/FEV1.

The lack of prediction equations for these and other novel parameters has limited quantitative analysis and interpretation of the shape of the flow-volume curve. This study provides prediction equations for novel spirometry parameters which allow a quantitative assessment of the flow-volume curve and an underpinning for future studies of the utility of these parameters with diagnosis of different pulmonary conditions.

To confirm that our selection criteria and analysis were comparable to that of Hankinson [4], regression equations for mean and lower limits of normal were compared. Both our analysis and that of Hankinson used age in months and had very similar regression equations, which were within 1% for all groups and parameters, with standard deviation under 2% for all parameters except for FEF25-75% using equations derived from both the 8-80 and 8-90 groups of subjects. Finding similar results supports the conclusion that our regression equations for the mean of other parameters and new parameters are valid. It is important to note that both our and Hankinson analysis used age in months/12 at time of exam rather than age in years, so age in months/12 or years + days/365 should be used in the spirometry regression equations.

While Hankinson [4] and Hansen [7] report separate regression equations for Caucasians and Mexican-American, Kiefer *et al*. [10] found similar spirometry values for Caucasians and Mexican-American and the GLI [5] group Caucasians with Mexican-American. We found that Mexican-American values were very similar to Caucasians, while values for Black differed.

Reference equation methods that account for non-linearity with age and can apply across all ages (3 to 90) have been described by Stanojevic [11] and used for GLI equations [5]. This study used methods similar to Hankinson and cannot be applied to subjects under age 8. While Hankinson uses one regression equation for age 8-80 in form b0+b1*age for each ratio parameter, the GLI [12] shows a plateau of FEV1/FVC from age 8 to 20, with an increased ratio below age 8. Therefore, it is preferable not to use a single linear equation for ratio parameters from age 8 to 90, with two linear equations fitting the data well. Equations derived from subjects up to age 90 were more appropriate to use for elderly patients. It should be noted that due to improvement in child health in the early 1900s, lung function of elderly subjects included in the NHANES study could differ from current elderly subjects and is a reason for continued studies of normative lung function.

We found that %predicted values were not normally distributed and had a similar variation for those under age 50 and those over age 50. While the equations for predicted values break near age 18 for females and 20 for males, the %predicted LLN and ULN equations break near age 50 with older subjects having more outliers. It is possible that some of the outliers had lost height or had lung or chest diseases unrelated to smoking.

There are many methods to determine equations for LLN and ULN. Prior spirometry analyses determined separate equations for each ethnicity/birth sex/age grouping for LLN for each spirometry parameter using an SAS function [4], GAMLSS package [5], or iterative method [7]. A study found differences in the classification of obstruction using GLI vs NHANES LLN parameters, mainly among older subjects [13]. The present study using an iterative method better matched the actual LLN than Hansen [7], which underestimates abnormalities in subjects below age 50 or Hankinson [4] which overestimates abnormalities in subjects above age 50. Our equations also fit the LLN for FVC better than those of Hankinson. Our finding that many parameters are not normally distributed and have different LLN z-scores than ULN z-scores shows that the ULN should not be calculated using LLN z-scores. While the 2022 ERS-ATS Interpretive Strategies for Routine Pulmonary Tests [9] recommends using z-scores to describe LLN and ULN, this study shows that %predicted values is an alternative method which has an advantage for parameters that are not normally distributed.

PFT results (spirometry, lung volume, DLCO) have been used to predict which patients have interstitial lung disease (ILD) [14]. Using new parameters such as PEF relative to FVC along with DACO (DLCO adjusted for lung volume) could improve screening for ILD [15]. Some novel parameters which may prove useful for the evaluation of ILD include PEF and %predicted PEF, so the validity of PEF measurements by NHANES is important. While PEF currently is generally measured using flow sensors, peak flow in NHANES was derived from the volume-time curve from a dry-rolling air seal spirometer using a digital filter and smoothing function. This method is felt to provide the same values as with other peak flow instruments [16].

The use of PFTs in evaluation of patient with interstitial lung disease (ILD) has been reviewed, but these do not include PEF relative to FVC [17]. In our experience, patients with early ILD often have a convex shaped flow-volume curve with FEV1 and FVC within normal limits but above normal PEF and elevated PEF and FEF25-75 relative to FVC. With early ILD, total lung capacity (TLC) and diffusing capacity of carbon monoxide (DLCO) may be near the lower limit of normal. As ILD progresses, FVC and PEF decrease, PEF further increases relative to FVC, TLC and alveolar volume (VA) decrease, and %predicted DLCO and DLCO/VA with predicted adjusted for lung volume (DACO and KACO) decrease. Unadjusted %predicted DLCO/VA (KCO) is often normal despite having low unadjusted and adjusted %predicted DLCO [18]. Further study is needed to determine the effectiveness of FEF25-75%/FVC, PEF/FVC, and DACO in diagnosing and better quantifying ILD severity.

Parameters involving FEV3 are of interest because many individuals are unable to expire at least 6 seconds, so interpretation must be made using FEV3 values. FEV1/FEV3 has been proposed as a valid measure indicating airflow obstruction, particularly in elderly patients who may be unable to cooperate with a 6-second exhalation [19]. FEV3/FEV6 has been advocated as an indicator of small airways disease, with current and former smokers having reduced FEV3/FEV6 but FEV1/FVC > 70% at increased risk of respiratory exacerbations and of developing FEV1/FVC < 70% [20].

## CONCLUSION

There is much more information in the flow-volume loop than the usual parameters, which could provide information on underlying lung disease. Using NHANES III data, this study provides prediction equations for mean, lower limit of normal, and upper limit of normal for existing and novel parameters for the age range of 8 to 90 among Caucasian/Mexican American and Black subjects. While mean values closely match prior studies, the lower limits of normal are better characterized with this study. Prediction equations for novel spirometry parameters, including FEF25-75%/FVC and PEF/FVC should allow a more comprehensive, quantitative evaluation of spirometry and flow-volume curves leading to improved detection of such processes as interstitial lung disease.

## Figures and Tables

**Fig. (1):**
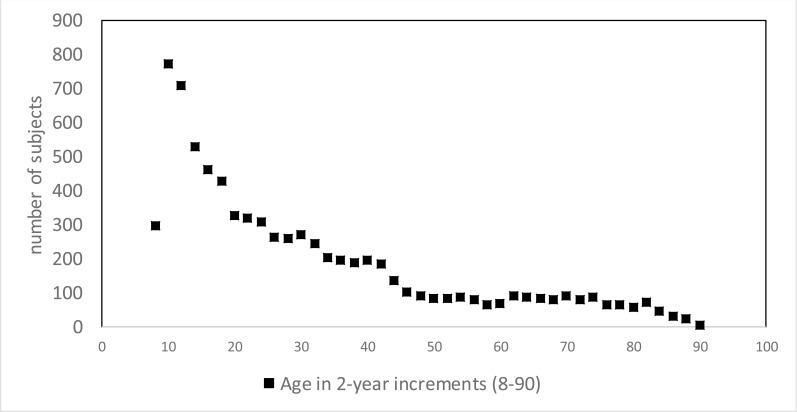
Number of subjects from age 8 to 90 by 2-year increments (>= 1 year below to <1 year above. n = 7851).

**Fig. (6):**
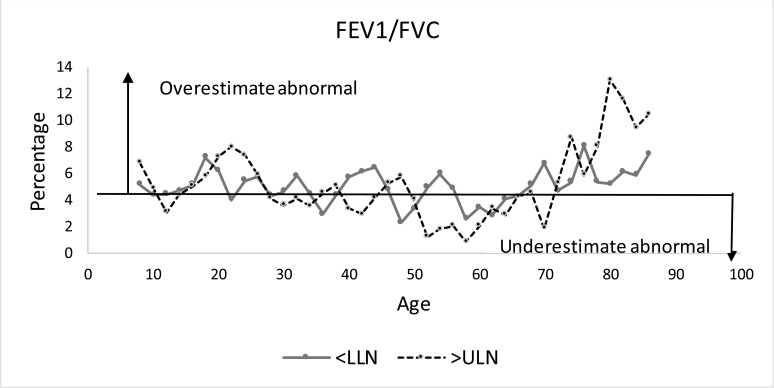
Percentage of subjects with FEV1/FVC below predicted lower limit of normal (LLN) or above predicted upper limit of normal (ULN) by age (those ± 2 years, overlapping 4-year intervals). Figure e2 has figures for all parameters.

**Fig. (7):**
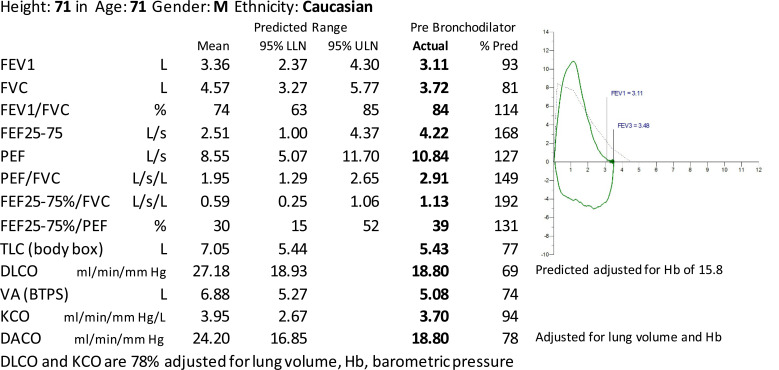
Spirometry in patient with interstitial lung disease confirmed by CT scan using predicted values from this study. FEV1 and FVC are within normal limits, PEF and FEF25-75% near lower limit of normal, and PEF/FVC and FEF25-75%/PEF above the upper limit of normal. TLC and DLCO are low, unadjusted KCO normal, and DLCO and KCO adjusted for lung volume 78% of predicted.

**Table 1a:** Prediction equations for non-ratio variables – male (8-90).

-	-	b0	b1	b2	b3	R^2^	SD
Caucasian/Mexican-American < 20 year of age (N = 1019)							
-	FEF25-75%	-0.208	-0.09687	0.0077037	0.00014180	0.640	1.338
-	FEF75%	-0.163	-0.08964	0.0045808	0.00008450	0.519	0.749
-	FEV05	-0.023	-0.09372	0.0061888	0.00009929	0.863	0.793
-	FEV1	-0.123	-0.15548	0.0087919	0.00014659	0.899	1.061
-	FEV3	-0.038	-0.20932	0.0110769	0.00017218	0.907	1.221
-	FEV6	-0.003	-0.21192	0.0112363	0.00017284	0.905	1.231
-	FVC	0.012	-0.21296	0.0112910	0.00017278	0.905	1.233
-	PEF	0.553	-0.33295	0.0220001	0.00025096	0.806	2.342
Caucasian/Mexican-American ≥ 20 year of age (N = 1130)							
-	FEF25-75%	3.707	-0.03441	-0.0001627	0.00006407	0.464	1.396
-	FEF75%	2.140	-0.06326	0.0003688	0.00003639	0.491	0.741
-	FEV05	0.920	0.00191	-0.0002283	0.00008489	0.564	0.618
-	FEV1	0.984	-0.01653	-0.0001309	0.00012975	0.670	0.815
-	FEV3	0.563	-0.02137	-0.0001038	0.00017052	0.682	0.931
-	FEV6	0.245	-0.01196	-0.0001723	0.00017890	0.653	0.927
-	FVC	-0.014	-0.00264	-0.0002275	0.00018221	0.609	0.905
-	PEF	1.047	0.07164	-0.0011848	0.00025889	0.379	1.946
Black < 20 year of age (N = 599)							
-	FEF25-75%	-0.553	-0.01181	0.0048576	0.00011409	0.554	1.333
-	FEF75%	-0.011	-0.07806	0.0047796	0.00005921	0.439	0.731
-	FEV05	-0.207	-0.07896	0.0047614	0.00009731	0.818	0.752
-	FEV1	-0.264	-0.14508	0.0071577	0.00013919	0.851	0.979
-	FEV3	-0.361	-0.18293	0.0082296	0.00016891	0.863	1.115
-	FEV6	-0.351	-0.18691	0.0083064	0.00017199	0.862	1.127
-	FVC	-0.345	-0.18819	0.0083586	0.00017247	0.862	1.130
-	PEF	-0.272	-0.23969	0.0167515	0.00025955	0.766	2.375
Black ≥20 year of age (N= 477)							
-	FEF25-75%	2.442	-0.00918	-0.0003896	0.00007423	0.277	1.326
-	FEF75%	2.003	-0.04776	0.0002398	0.00002450	0.304	0.713
-	FEV05	0.620	-0.00642	-0.0001309	0.00008605	0.384	0.586
-	FEV1	0.825	-0.02465	-0.0000085	0.00011813	0.476	0.722
-	FEV3	0.684	-0.03150	0.0000508	0.00014534	0.499	0.796
-	FEV6	0.469	-0.02688	0.0000273	0.00015267	0.470	0.800
-	FVC	0.243	-0.02183	0.0000037	0.00015761	0.437	0.800
-	PEF	1.316	0.00498	-0.0005885	0.00027615	0.267	2.047^1^

**Note:** Predicted lung function parameter = b0 + b1 * age + b2*age^2^ + b3*height^2^. Age in years (including fraction of years). Height in centimeters.

**Table 1b:** Prediction equations for non-ratio variables – female (8-90).

-	-	b0	b1	b2	b3	R^2^	SD
Caucasian/Mexican-American < 18 year of age (N = 966)							
-	FEF25-75%	-2.287	0.36871	-0.0105031	0.00011163	0.494	0.980
-	FEF75%	-0.771	0.04738	-0.0001678	0.00007382	0.416	0.601
-	FEV05	-1.118	0.14061	-0.0034854	0.00008481	0.754	0.523
-	FEV1	-1.261	0.09815	-0.0014094	0.00012443	0.820	0.693
-	FEV3	-1.237	0.06806	-0.0001246	0.00014304	0.826	0.774
-	FEV6	-1.172	0.05717	0.0002509	0.00014467	0.821	0.779
-	FVC	-1.173	0.05817	0.0002240	0.00014468	0.821	0.780
-	PEF	-4.373	0.67357	-0.0201231	0.00020547	0.627	1.521
Caucasian/Mexican-American ≥ 18 year of age (N = 2113)							
-	FEF25-75%	3.122	-0.02647	-0.0001425	0.00005124	0.534	1.147
-	FEF75%	1.971	-0.05319	0.0003013	0.00002872	0.534	0.655
-	FEV05	0.692	0.00282	-0.0001934	0.00007177	0.643	0.499
-	FEV1	0.777	-0.00921	-0.0001374	0.00010647	0.720	0.655
-	FEV3	0.438	-0.00690	-0.0001700	0.00013529	0.709	0.729
-	FEV6	0.210	0.00025	-0.0002216	0.00014105	0.676	0.721
-	FVC	0.029	0.00588	-0.0002559	0.00014407	0.641	0.705
-	PEF	1.029	0.05084	-0.0008555	0.00019732	0.422	1.467
Black < 18 year of age (N = 590)							
-	FEF25-75%	-1.360	0.12216	-0.0012925	0.00012258	0.446	1.014
-	FEF75%	-0.437	-0.01396	0.0023239	0.00006395	0.360	0.582
-	FEV05	-0.800	0.07436	-0.0009263	0.00007802	0.720	0.490
-	FEV1	-1.097	0.07939	-0.0008047	0.00010671	0.764	0.620
-	FEV3	-1.112	0.07110	-0.0005303	0.00012040	0.763	0.675
-	FEV6	-1.089	0.06843	-0.0004901	0.00012183	0.759	0.678
-	FVC	-1.051	0.06463	-0.0003505	0.00012161	0.757	0.677
-	PEF	-3.286	0.49615	-0.0123598	0.00018946	0.597	1.506
Black ≥18 year of age (N= 957)							
-	FEF25-75%	2.798	-0.04689	0.0000817	0.00006712	0.384	1.135
-	FEF75%	1.813	-0.05565	0.0003690	0.00002760	0.415	0.614
-	FEV05	0.551	-0.00803	-0.0000875	0.00007530	0.511	0.476
-	FEV1	0.455	-0.01787	-0.0000341	0.00010810	0.596	0.590
-	FEV3	0.045	-0.01405	-0.0000784	0.00013424	0.585	0.650
-	FEV6	-0.181	-0.00723	-0.0001318	0.00013999	0.554	0.647
-	FVC	-0.320	-0.00221	-0.0001663	0.00014210	0.523	0.638
-	PEF	1.903	0.02095	-0.0006620	0.00018594	0.307	1.562^2^

**Note:** Predicted lung function parameter = b0 + b1 * age + b2*age^2^ + b3*height^2^. Age in years (including fraction of years). Height in centimeters.

**Table 2a:** Prediction equations for ratio variables - male 8-90.

-	-	b0	b1	R^2^	SD
Caucasian/Mexican-American < 20 year of age (N = 1019)					
-	FEF25-75%/FVC	1.059	-0.0056	0.006	0.240
-	FEF25-75%/PEF	58.059	-0.3135	0.010	10.117
-	FEF75%/FVC	43.997	0.0596	0.000	15.638
-	FEF75%/PEF	24.405	0.0162	0.000	8.179
-	FEV05/FEV3	71.878	-0.3196	0.025	6.627
-	FEV05/FVC	70.074	-0.2677	0.016	6.948
-	FEV1/FEV3	89.603	-0.1150	0.006	4.887
-	FEV1/FEV6	87.754	-0.0665	0.002	5.525
-	FEV1/FVC	87.300	-0.0513	0.001	5.636
-	FEV3/FEV6	97.881	0.0536	0.015	1.454
-	FEV3/FVC	97.360	0.0709	0.018	1.725
-	FEV6/FVC	99.466	0.0178	0.010	0.591
-	PEF/FEV1	2.091	0.0013	0.000	0.275
-	PEF/FEV6	1.836	-0.0001	0.000	0.288
-	PEF/FVC	1.826	0.0002	0.000	0.288
Caucasian/Mexican-American ≥ 20 year of age (N = 1130)					
-	FEF25-75%/FVC	1.075	-0.0068	0.228	0.260
-	FEF25-75%/PEF	56.780	-0.3790	0.317	12.257
-	FEF75%/FVC	45.832	-0.4383	0.307	14.384
-	FEF75%/PEF	24.252	-0.2360	0.318	7.613
-	FEV05/FEV3	67.031	0.0117	0.001	6.434
-	FEV05/FVC	67.762	-0.1077	0.071	7.335
-	FEV1/FEV3	88.255	-0.0630	0.064	4.538
-	FEV1/FEV6	87.881	-0.1355	0.179	5.832
-	FEV1/FVC	89.029	-0.2101	0.293	7.058
-	FEV3/FEV6	99.721	-0.0904	0.434	2.497
-	FEV3/FVC	101.185	-0.1817	0.499	4.680
-	FEV6/FVC	101.663	-0.0997	0.429	2.771
-	PEF/FEV1	2.107	0.0073	0.133	0.365
-	PEF/FEV6	1.872	0.0027	0.022	0.333
-	PEF/FVC	1.905	0.0007	0.002	0.329
Black < 20 year of age (N = 599)					
-	FEF25-75%/FVC	1.045	-0.0018	0.000	0.287
-	FEF25-75%/PEF	51.284	-0.1295	0.002	10.528
-	FEF75%/FVC	39.781	0.3217	0.004	17.871
-	FEF75%/PEF	19.477	0.1415	0.004	7.647
-	FEV05/FEV3	73.201	-0.2265	0.012	7.172
-	FEV05/FVC	71.206	-0.1770	0.006	7.671
-	FEV1/FEV3	89.225	-0.0421	0.001	5.329
-	FEV1/FEV6	87.122	0.0107	0.000	6.134
-	FEV1/FVC	86.746	0.0159	0.000	6.285
-	FEV3/FEV6	97.590	0.0583	0.014	1.698
-	FEV3/FVC	97.164	0.0638	0.012	1.994
-	FEV6/FVC	99.563	0.0055	0.001	0.621
-	PEF/FEV1	2.329	0.0020	0.001	0.299
-	PEF/FEV6	2.032	0.0022	0.001	0.332
-	PEF/FVC	2.023	0.0023	0.001	0.334
Black ≥20 year of age (N= 477)					
-	FEF25-75%/FVC	1.111	-0.0064	0.112	0.278
-	FEF25-75%/PEF	51.249	-0.2701	0.124	11.110
-	FEF75%/FVC	48.851	-0.4627	0.177	15.919
-	FEF75%/PEF	22.613	-0.2046	0.163	7.323
-	FEV05/FEV3	69.097	0.0063	0.000	7.214
-	FEV05/FVC	69.653	-0.1045	0.035	8.069
-	FEV1/FEV3	88.816	-0.0565	0.026	5.098
-	FEV1/FEV6	88.504	-0.1287	0.087	6.312
-	FEV1/FVC	89.359	-0.1913	0.151	7.113
-	FEV3/FEV6	99.680	-0.0867	0.282	2.363
-	FEV3/FVC	100.709	-0.1610	0.353	3.919
-	FEV6/FVC	101.140	-0.0799	0.292	2.139
-	PEF/FEV1	2.400	0.0046	0.030	0.383
-	PEF/FEV6	2.134	0.0007	0.001	0.382
-	PEF/FVC	2.158	-0.0010	0.002	0.382^3^

Predicted lung function parameter = b0 + b1 * age. Age in years (including fraction of years). Height in centimeters. Ratio of flows or of volumes in percent; ratio of flow/volume in L/min/L.

**Table 2b:** Prediction equations for ratio variables - female 8-90.

-		b0	b1	R^2^	SD
Caucasian/Mexican-American < 18 year of age (N = 966)					
-	FEF25-75%/FVC	1.202	-0.0076	0.007	0.255
-	FEF25-75%/PEF	58.309	0.0010	0.000	10.519
-	FEF75%/FVC	46.537	0.3862	0.004	16.799
-	FEF75%/PEF	22.292	0.3985	0.016	8.949
-	FEV05/FEV3	76.192	-0.4317	0.030	6.972
-	FEV05/FVC	74.665	-0.3772	0.022	7.188
-	FEV1/FEV3	90.868	-0.0469	0.001	4.711
-	FEV1/FEV6	89.300	0.0088	0.000	5.218
-	FEV1/FVC	88.991	0.0168	0.000	5.305
-	FEV3/FEV6	98.241	0.0613	0.017	1.325
-	FEV3/FVC	97.910	0.0690	0.016	1.535
-	FEV6/FVC	99.665	0.0076	0.002	0.461
-	PEF/FEV1	2.311	-0.0144	0.019	0.292
-	PEF/FEV6	2.068	-0.0128	0.014	0.305
-	PEF/FVC	2.061	-0.0126	0.014	0.304
Caucasian/Mexican-American ≥ 18 year of age (N = 2113)					
-	FEF25-75%/FVC	1.195	-0.0074	0.234	0.305
-	FEF25-75%/PEF	64.557	-0.4210	0.335	14.402
-	FEF75%/FVC	54.149	-0.5276	0.309	18.804
-	FEF75%/PEF	29.483	-0.2941	0.350	9.849
-	FEV05/FEV3	69.255	0.0001	0.000	6.656
-	FEV05/FVC	70.424	-0.1138	0.087	7.654
-	FEV1/FEV3	90.133	-0.0781	0.106	4.746
-	FEV1/FEV6	90.359	-0.1544	0.242	6.213
-	FEV1/FVC	91.465	-0.2168	0.330	7.481
-	FEV3/FEV6	100.405	-0.0933	0.466	2.708
-	FEV3/FVC	101.786	-0.1687	0.478	4.832
-	FEV6/FVC	101.545	-0.0821	0.357	2.722
-	PEF/FEV1	1.986	0.0075	0.142	0.395
-	PEF/FEV6	1.819	0.0027	0.022	0.351
-	PEF/FVC	1.849	0.0010	0.004	0.347
Black < 18 year of age (N = 590)					
-	FEF25-75%/FVC	1.067	0.0042	0.001	0.314
-	FEF25-75%/PEF	48.716	0.3384	0.007	11.151
-	FEF75%/FVC	40.506	0.7691	0.012	19.860
-	FEF75%/PEF	17.952	0.4659	0.023	8.637
-	FEV05/FEV3	72.419	-0.0144	0.000	7.825
-	FEV05/FVC	70.335	0.0686	0.001	8.398
-	FEV1/FEV3	87.727	0.1990	0.009	5.911
-	FEV1/FEV6	85.695	0.2765	0.013	6.692
-	FEV1/FVC	85.074	0.3049	0.015	6.993
-	FEV3/FEV6	97.526	0.0985	0.024	1.786
-	FEV3/FVC	96.711	0.1382	0.024	2.525
-	FEV6/FVC	99.095	0.0456	0.011	1.213
-	PEF/FEV1	2.495	-0.0103	0.007	0.337
-	PEF/FEV6	2.161	-0.0036	0.001	0.372
-	PEF/FVC	2.148	-0.0030	0.001	0.375
Black ≥18 year of age (N= 957)					
-	FEF25-75%/FVC	1.239	-0.0075	0.151	0.325
-	FEF25-75%/PEF	58.565	-0.3579	0.203	13.327
-	FEF75%/FVC	54.312	-0.5219	0.209	19.162
-	FEF75%/PEF	25.840	-0.2491	0.219	8.927
-	FEV05/FEV3	72.370	-0.0255	0.004	6.882
-	FEV05/FVC	73.170	-0.1347	0.080	7.965
-	FEV1/FEV3	91.109	-0.0830	0.086	4.751
-	FEV1/FEV6	91.227	-0.1593	0.186	6.197
-	FEV1/FVC	92.023	-0.2156	0.249	7.237
-	FEV3/FEV6	100.231	-0.0915	0.328	2.679
-	FEV3/FVC	101.221	-0.1588	0.373	4.361
-	FEV6/FVC	101.105	-0.0728	0.267	2.36
-	PEF/FEV1	2.290	0.0064	0.063	0.426
-	PEF/FEV6	2.104	0.0014	0.004	0.397
-	PEF/FVC	2.126	-0.0001	0.000	0.398^4^

**Note:** Predicted lung function parameter = b0 + b1 * age. Age in years (including fraction of years). Height in centimeters. Ratio of flows or of volumes in percent; ratio of flow/volume in L/min/L.

**Table 3:** % Predicted lower limit of normal (LLN), upper limit of normal (ULN), standard deviation (SD), and z-scores. For all subjects, those under age 50, and those 50 and above.

-	-	All (n = 7851)		-	-	-	< age 50 (n=6460)			-	-	≥ age 50 (n = 1391)			-
	LLN	ULN	SD	LLNz	ULNz	LLN	ULN	SD	LLNz	ULNz	LLN	ULN	SD	LLNz	ULNz
FEF25-75%	57.7	147.7	28.62	-1.479	1.668	61.5	143.5	25.22	-1.525	1.727	40.0	174.1	41.24	-1.455	1.796
FEF75%	46.2	174.3	44.36	-1.213	1.675	50.8	166.5	36.59	-1.344	1.816	31.8	218.9	70.64	-0.966	1.683
FEV05	76.9	122.9	14.42	-1.605	1.587	78.7	121.5	13.26	-1.604	1.624	66.5	128.7	19.03	-1.762	1.510
FEV1	78.8	121.2	13.20	-1.605	1.603	80.7	119.6	12.01	-1.605	1.632	70.6	127.9	17.85	-1.646	1.563
FEV3	79.7	120.6	12.78	-1.588	1.613	81.0	119.2	11.74	-1.623	1.633	72.4	127.5	16.92	-1.629	1.627
FEV6	79.9	120.3	12.76	-1.579	1.592	81.1	119.2	11.77	-1.607	1.629	72.6	127.2	16.70	-1.641	1.631
FVC	79.8	120.6	12.83	-1.574	1.604	81.0	119.3	11.78	-1.613	1.636	71.6	126.4	17.01	-1.671	1.551
PEFR	70.8	129.7	18.10	-1.613	1.642	72.9	127.5	16.89	-1.604	1.629	59.3	136.8	23.04	-1.768	1.598
FEF25-75%/FVC	56.8	149.6	30.02	-1.438	1.651	61.0	145.3	26.24	-1.487	1.728	42.8	179.5	43.86	-1.303	1.813
FEF25-75%/PEFR	64.2	139.9	24.98	-1.435	1.597	68.1	135.1	20.73	-1.538	1.692	49.5	175.6	39.50	-1.278	1.915
FEF75%/FVC	45.6	178.3	54.82	-0.992	1.429	49.5	168.8	38.95	-1.297	1.766	34.4	282.6	100.37	-0.654	1.819
FEF75%/PEFR	46.5	171.5	52.69	-1.016	1.356	50.0	159.4	35.62	-1.403	1.667	35.5	289.7	99.01	-0.651	1.915
FEV05/FEV3	84.3	116.4	10.03	-1.566	1.639	84.3	116.7	10.04	-1.562	1.666	83.9	114.1	9.94	-1.621	1.422
FEV05/FVC	82.1	118.4	11.31	-1.582	1.623	83.0	118.1	10.93	-1.553	1.656	78.9	120.5	12.94	-1.631	1.581
FEV1/FEV3	90.7	108.3	5.44	-1.703	1.533	90.7	108.5	5.43	-1.719	1.564	91.3	107.4	5.45	-1.596	1.357
FEV1/FEV6	89.0	110.1	6.58	-1.680	1.542	89.1	110.0	6.38	-1.714	1.561	88.5	111.0	7.44	-1.546	1.474
FEV1/FVC	87.8	111.1	7.29	-1.674	1.529	88.5	110.5	6.73	-1.712	1.560	84.7	114.9	9.53	-1.609	1.565
FEV3/FEV6	96.9	102.5	1.89	-1.620	1.295	97.1	102.2	1.66	-1.720	1.326	96.1	105.6	2.74	-1.432	2.052
FEV3/FVC	94.8	104.1	3.11	-1.658	1.306	96.0	103.1	2.33	-1.707	1.310	91.1	110.1	5.49	-1.619	1.830
FEV6/FVC	97.1	102.4	1.74	-1.681	1.364	98.2	101.4	1.05	-1.743	1.353	93.8	105.1	3.50	-1.760	1.442
PEFR/FEV1	77.0	123.7	14.46	-1.592	1.638	78.3	123.0	13.75	-1.577	1.670	70.7	128.3	17.46	-1.680	1.623
PEFR/FEV6	73.6	128.8	17.04	-1.551	1.687	74.5	128.2	16.41	-1.556	1.716	67.4	132.6	19.81	-1.644	1.648
PEFR/FVC	73.0	129.4	17.32	-1.559	1.699	74.3	128.5	16.54	-1.552	1.726	66.0	135.6	20.66	-1.643	1.725

## Data Availability

Not applicable.

## LIST OF ABBREVIATIONS

Predicted DACO: = Predicted DLCO also adjusted for lung volume
DLCO: = Diffusing capacity of carbon monoxide
FEF25–75%: = Forced expiratory flow at 25–75% of FVC
FEF75%: = The flow at 75% of FVC
FEV05, FEV1, FEV3, FEV6: = The expiratory volume in the first 0.5, 1, 3, or 6 seconds of a FVC maneuver
FVC: = Forced vital capacity
GLI: = Global Lung Initiative;
LLN: = Lower limit of normal
Predicted KACO: = Predicted KCO also adjusted for lung volume
KCO: = DLCO/ VA
ILD: = Interstitial lung disease
NHANES: = National Health and Nutrition Examination Survey
PEF: = Peak expiratory flow
PFT: = Pulmonary function testing
SD: = Standard deviation
TLC: = Total lung capacity
ULN: = Upper limit of normal
VA: = Alveolar volume
