# Supplementary material for: Spirometry Reference Equations Including Existing and Novel Parameters
Source: Open Respir Med J. 2023 Feb 23;17:e187430642212260. doi: 10.2174/18743064-v16-e221227-2022-14 (PMC10351349; doi:10.2174/18743064-v16-e221227-2022-14)
Supplement: Supplementary file 1 — Supplementary material is available on the Publisher’s website. [file TORMJ-17-E187430642212260_SP.pdf]

1874-3064/23 Send Orders for Reprints to reprints@benthamscience.net

The Open Respiratory Medicine Journal

Content list available at: https://openrespiratorymedicinejournal.com

Spirometry Reference Equations Including Existing and Novel Parameters

Douglas Clark Johnson[1,*] and Bradford Gardner Johnson[2]

[1]*Department of Medicine, Baystate Medical Center 759 Chestnut Street Springfield, MA 01199, USA*
[2]*Washington, D.C. 20010 USA*

Article History	Received: August 10, 2022	Revised: October 9, 2022	Accepted: November 4, 2022

APPENDIX

Of the 16840 adult and 4146 youth who performed spirometry, 4863 (29%) adults age 17- 80, 5073 (30%) adults age 17-90 (Appendix Table 1), and 2779 (52%) youth age 8 to 17 (Appendix Table 2) remained to be analyzed after applying the exclusion criteria including python code with NHANES III database field names.

Regression equations from this study were very close to those of Hankinson (Appendix Table 3). For the all subject group the difference was < 1% for all parameters, with standard deviation < 2% for all parameters except for FEF25-75% and PEF. NHANES includes 210 subjects age 80 to 90 who otherwise meet inclusion criteria. While Hankinson in general used equations in form $b0+b1*age+b2*age^2+b3*height^2$, for FEF25-75% b2 was set to 0. Using b2 of 0 improved the prediction equations over those derived from the 8-80 group, and closer to the regressions determined from this study derived from the 8-90 group.

Blacks had 15% lower FEV1, 16% lower FVC, 11% lower FEF25-75%, identical FEV1/FVC, 5% lower PEF, and 11% higher PEF/FVC as Caucasian/Mexican-American (Appendix Table 4).

Using Hankinson equations for LLN, most parameters had subgroups with <4% or > 6% of subjects below the LLN, ranging from 2.2% to 8.0% (Appendix Table 5a). Using Hansen's equations, adult subgroups had from 3.4% to 6.3% of subjects below the LLN (Appendix Table 5b).

Appendix Table 1. Total normative adult subjects below 90 years 5072.

Excluded		Remaining	Criteria of those Remaining
Total in adult exam file	-	20050	-
Not have spirometry	3210	16840	SPPMANEU != 88
Fewer than 2 acceptable curves	370	16470	SPPMANEU > 1 & SPPMANEU < 88
Unreproducible FEV1	181	16289	SPPREPRO == 4 | SPPREPRO == 1
90 year of age (1080 month) or older	262	16027	MXPAXTMR < 1080
Race-ethnicity coded as "Other"	632	15395	DMARETHN < 4
Cigarette smokers	7,631	7764	not HAR1 ==1
Cigar and/or pipe smokers	310	7454	not HAR23 == 1 not HAR26 == 1
Smoked cigarettes, cigars, or pipes in prior 5 days	0	7454	not (0 < HXPG1 < 888 | 0 < HXPG2A < 88 | 0 < HXPG2B < 88)
Physician diagnosis of asthma	467	6987	not HAR26 == 1
Physician diagnosis of chronic bronchitis	190	6797	not HAC1E == 1
Physician diagnosis of emphysema	15	6782	not HAC1F == 1
Physician diagnosis of lung cancer	0	6782	not HAC1G == 1
Whistling and/or wheezing in chest in last 12 months	441	6341	not HAL6 == 1
Whistling and/or wheezing, apart from colds	121	6220	not HAL10 == 1
Persistent cough	161	6059	not HAL1 == 1
Persistent phlegm production	129	5930	not HAL3 == 1
Moderate shortness of breath	857	5073	not HAL5 == 1

DOI: 10.2174/18743064-v16-e221227-2022-14, 2023, *17*, e187430642212260

Excluded		Remaining	Criteria of those Remaining
Have invalid FEV3	1	5072	not SPPFEV3 == 8888
80 yr of age (960 month) or older	209	4863	MXPAXTMR < 960

Appendix Table **1**. Number of subjects from adult file excluded using exclusion criteria. The criteria of those remaining include the python code with NHANES III database field names. Age was determined from age in months at the time of spirometry exam.

Appendix Table 2. Total normative youth subjects 2779.

-	excluded	remaining	criteria of those remaining
Total in youth exam file	-	13944	-
Not have spirometry *	9798	4146	SPPMANEU > -1 & SPPMANEU < 88
Less than 2 acceptable curves	32	4114	SPPMANEU > 1 & SPPMANEU < 88
Unreproducible FEV1	38	4076	SPPREPRO == 4 | SPPREPRO == 1
Race/ethnicity coded as “Other”	186	3890	DMARETHN < 4
Cigarette smokers	115	3775	not MYPB3 == 1
Smoked cigarettes, cigars, and/or pipes in prior 5 days	220	3555	not MYPB11 >0 not MYPB27A >0
Physician diagnosis of asthma	323	3232	not HYE1G == 1
Physician diagnosis of chronic bronchitis	87	3145	not HYE1H == 1
Whistling and/or wheezing in chest in last 12 month	278	2867	not HYG8 == 1
Whistling and/or wheezing in chest, apart from colds	51	2816	not HYG12 == 1
Persistent cough	16	2800	not HYG2 == 1
Persistent phlegm production	10	2790	not HYG4 == 1
constant “problems” with coughing in prior 12 month	10	2780	not HYG7 == 555
Invalid height	1	2779	BMPHT > 114

Appendix Table **2**. Number of subjects from youth file excluded using exclusion criteria. The criteria of those remaining include the python code with NHANES III database variable names. *all subjects with spirometry were age 8 (96 month) to age 17 year 3 month.

Appendix Table 3.

-	Caucasian		Black		Mexican-American		All	
-	N = 2464		N = 2623		N = 2764		N = 7851	
-	mean	SD	mean	SD	mean	SD	mean	SD
FEV1 PPdiff	-0.081	2.539	0.268	1.795	0.204	1.419	0.136	1.957
FEV6 PPdiff	-0.214	2.095	-0.041	1.880	0.130	1.214	-0.035	1.760
FVC PPdiff	-0.101	1.658	-0.056	1.829	0.127	1.121	-0.006	1.560
FEF25-75% PPdiff	-0.537	6.866	1.219	3.855	0.462	3.323	0.401	4.913
PEF PPdiff	0.380	2.003	0.546	2.504	0.310	1.526	0.411	2.045
FEV1/FVC PPdiff	-0.157	0.541	0.072	0.878	-0.008	0.462	-0.028	0.658
FEV1/FEV6 PPdiff	-0.069	0.562	0.092	0.721	0.027	0.467	0.019	0.595

Appendix Table **3**. Difference in %predicted values using regression equations from this study minus those using Hankinson equations. N = number of subjects.

Appendix Table 4.

-	BM≥20	BF≥18	BM<20	BF<18	average
FEF75%	91.5	87.7	82.0	83.0	86.0
FEV05	87.2	88.1	87.0	87.7	87.5
FEV1	85.1	85.6	84.1	85.3	85.0
FEV3	83.9	84.4	83.4	85.2	84.2
FEV6	83.8	84.5	83.6	85.4	84.3
FVC	83.6	84.6	83.7	85.5	84.3
FEF25-75%	90.8	90.7	87.3	87.6	89.1
PEF	92.8	96.4	94.6	95.8	94.9
FEF75%/FVC	107.3	103.2	98.4	97.6	101.6

-	BM≥20	BF≥18	BM<20	BF<18	average
FEF75%/PEF	97.9	92.1	86.8	87.2	91.0
FEV05/FEV3	102.8	103.1	103.8	102.1	103.0
FEV05/FVC	103.1	102.9	103.5	101.9	102.9
FEV1/FVC	101.3	100.7	100.4	99.7	100.5
FEV1/FEV3	100.9	100.9	100.7	100.0	100.6
FEV1/FEV6	101.0	100.8	100.5	99.7	100.5
FEV3/FEV6	100.1	99.9	99.8	99.8	99.9
FEV6/FVC	100.2	99.9	99.9	99.9	100.0
FEF25-75%/FVC	106.2	104.5	103.8	101.4	104.0
FEF25-75%/PEF	96.8	92.9	92.0	90.9	93.1
PEF/FEV1	108.3	111.6	111.8	111.1	110.7
PEF/FEV6	109.6	112.5	112.4	111.0	111.4
PEF/FVC	109.8	112.5	112.3	111.0	111.4

Appendix Table **4**. Black % predicted relative to %predicted Caucasian/Mexican American as percent for groups male 20 and over (BM≥20), female 18 and over (BF≥18), male less than 20 (BM<20), and female less than 18 (BF<18).

Appendix Table 5a.

-	m<20b	m<20c	m<20ma	m≥20b	m≥20c	m≥20ma
FEV1	4.0	2.2	4.7	6.9	6.6	5.7
FEV6	3.3	2.6	3.8	5.7	5.3	6.6
FVC	3.3	2.4	3.7	6.1	5.1	6.0
FEF25-75%	2.5	2.4	4.3	5.0	4.2	5.0
PEF	3.7	3.1	2.7	8.0	5.9	5.7
FEV1/FEV6	4.7	6.0	5.5	5.7	5.7	5.2
FEV1/FVC	4.2	4.5	4.7	5.9	6.1	5.7
n	599	419	600	477	528	602
-	**f<18b**	**f<18c**	**f<18ma**	**f≥18b**	**f≥18c**	**f≥18ma**
FEV1	2.7	3.7	4.9	4.7	5.2	5.2
FEV6	2.9	2.7	3.4	4.5	6.2	4.4
FVC	2.7	2.4	3.1	5.1	6.5	4.5
FEF25-75%	6.4	6.4	7.0	3.9	3.9	5.9
PEF	3.4	5.1	4.1	6.1	5.8	5.7
FEV1/FEV6	7.6	4.9	5.0	4.0	5.8	5.5
FEV1/FVC	7.0	4.4	4.7	4.3	6.1	5.8
n	590	409	557	957	1108	1005
n male + female	1189	828	1157	1434	1636	1607

Appendix Table **5a**. % of patients below Hankinson LLN

Appendix Table 5b.

	m≥20b	m≥20c	m≥20ma	f≥18b	f≥18c	f≥18ma
FEV1/FEV6	3.6	6.1	4.8	4.4	5.5	4.9
FEV1/FVC	3.4	6.3	4.5	4.5	5	5.4
FEV3/FEV6	5	6.3	4.7	4.8	4.4	5.2
FEV3/FVC	3.6	5.7	4.5	6.1	3.9	4.3

Appendix Table **5b**. % of patients below Hansen LLN

Appendix Table 5. % of subjects below Hankinson (5a) and Hansen (5b) lower limit of normal (LLN). m<20 – male age less than 20; m≥20 - male age 20 and over; f<18 – female age less than 18; f≥18 – female age 18 and over; b – Black; c – Caucasian; ma – Mexican American.

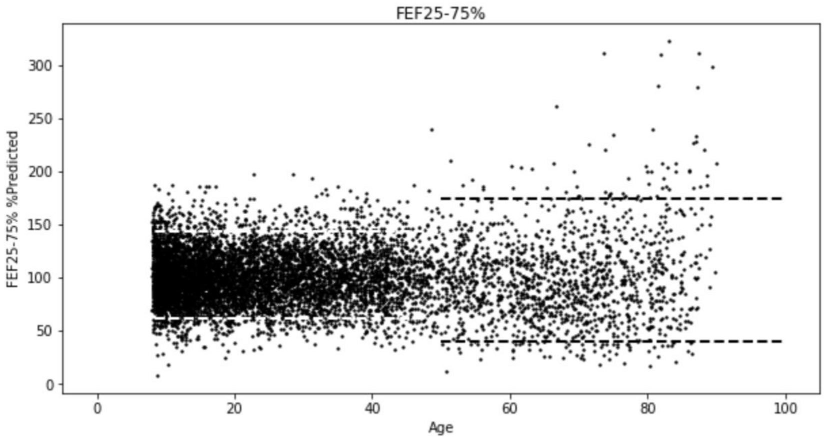
FEF25-75%
FEF25-75% %Predicted
Age
0
50
100
150
200
250
300
0
20
40
60
80
100

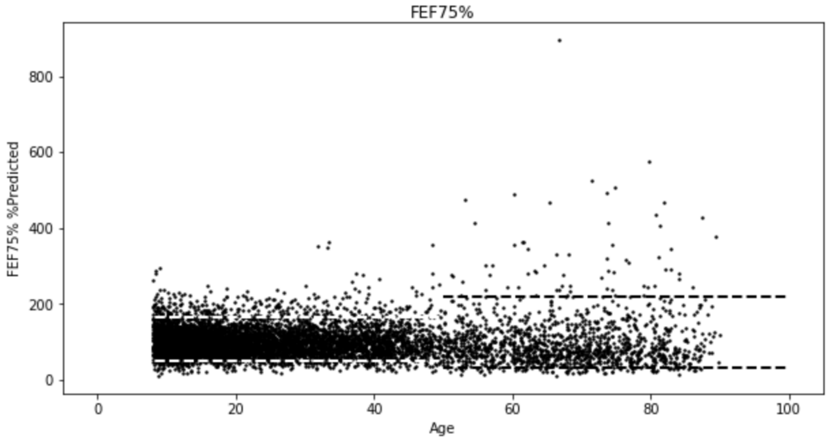
FEF75%
FEF75% %Predicted
Age
0
200
400
600
800
0
20
40
60
80
100

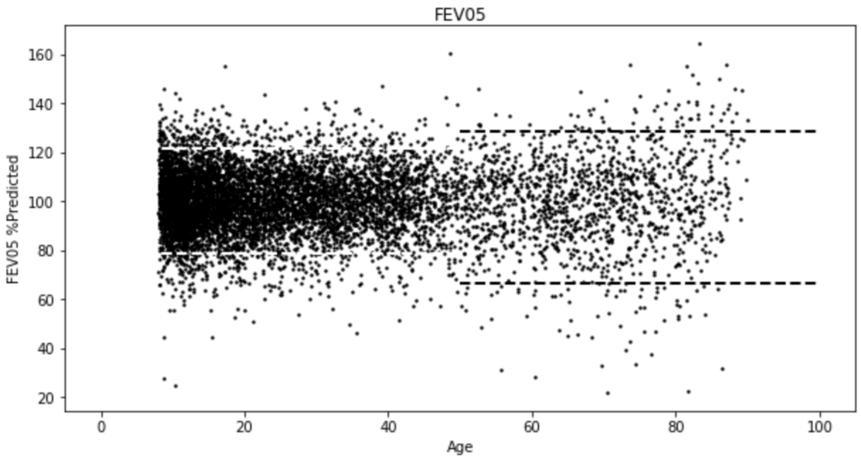
FEV05
FEV05 %Predicted
Age
20
40
60
80
100
120
140
160
0
20
40
60
80
100

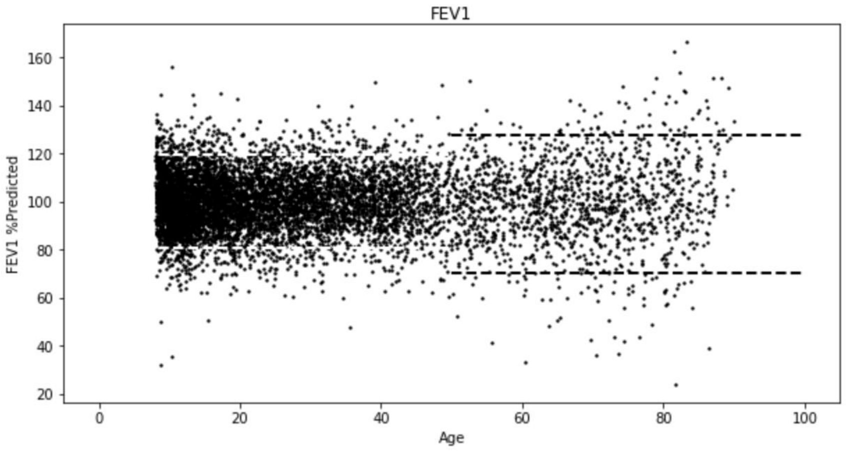
FEV1
FEV1 %Predicted
Age
0
20
40
60
80
100
160
140
120
100
80
60
40
20

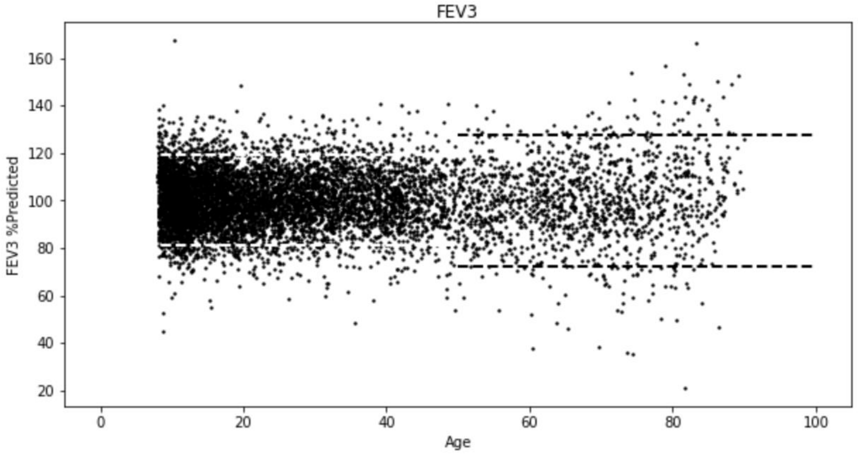
FEV3
FEV3 %Predicted
Age
0
20
40
60
80
100
160
140
120
100
80
60
40
20

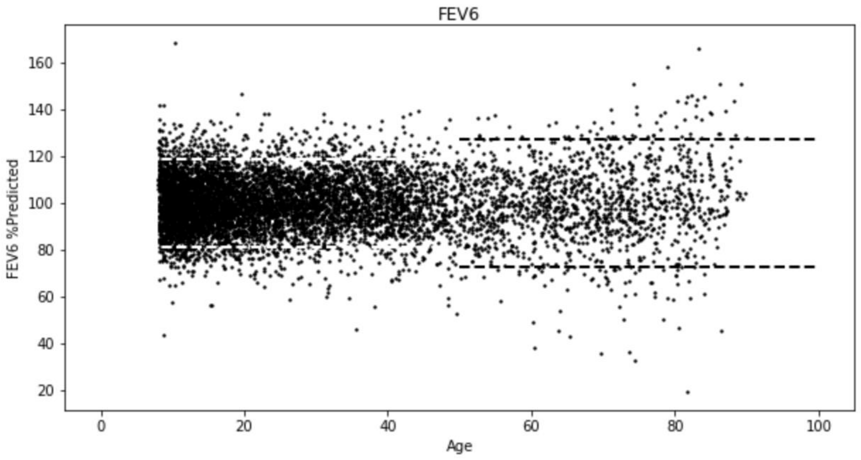
FEV6
FEV6 %Predicted
Age
0
20
40
60
80
100
160
140
120
100
80
60
40
20

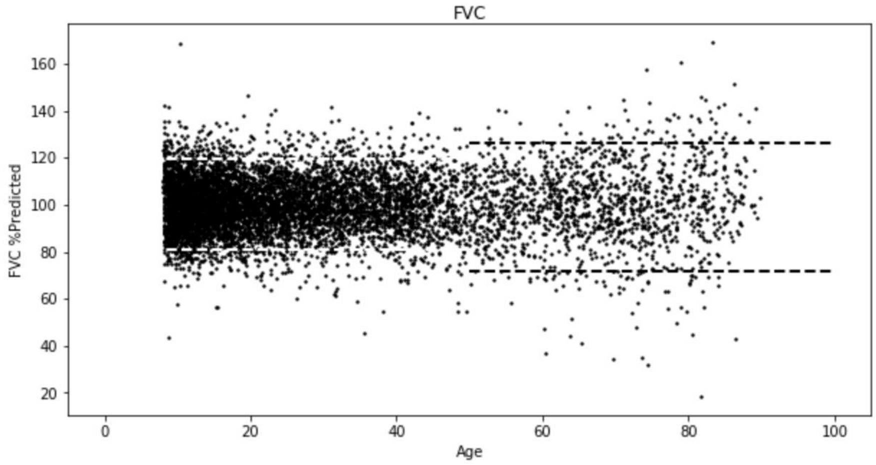
FVC
FVC %Predicted
Age
0
20
40
60
80
100
20
40
60
80
100
120
140
160

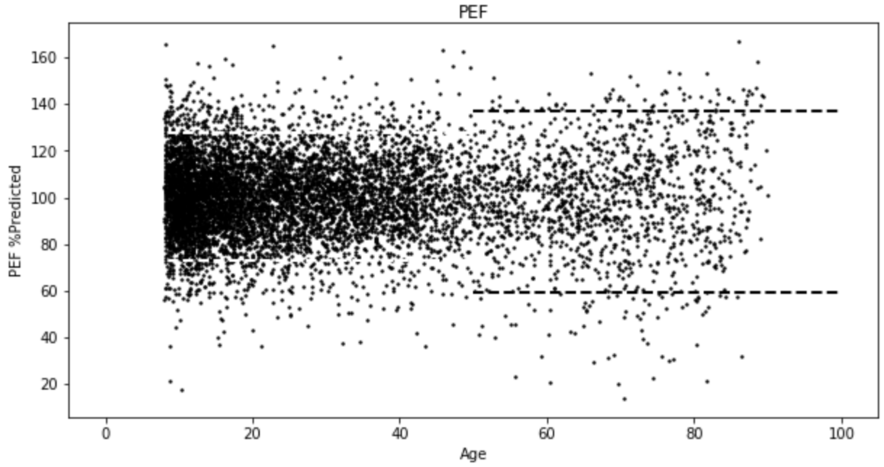
PEF
PEF %Predicted
Age
0
20
40
60
80
100
20
40
60
80
100
120
140
160

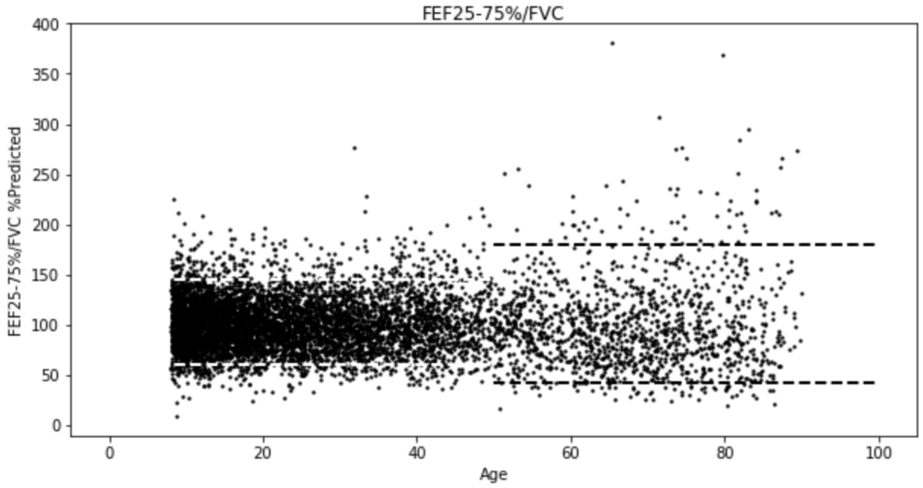
FEF25-75%/FVC
FEF25-75%/FVC %Predicted
Age
0
20
40
60
80
100
0
50
100
150
200
250
300
350
400

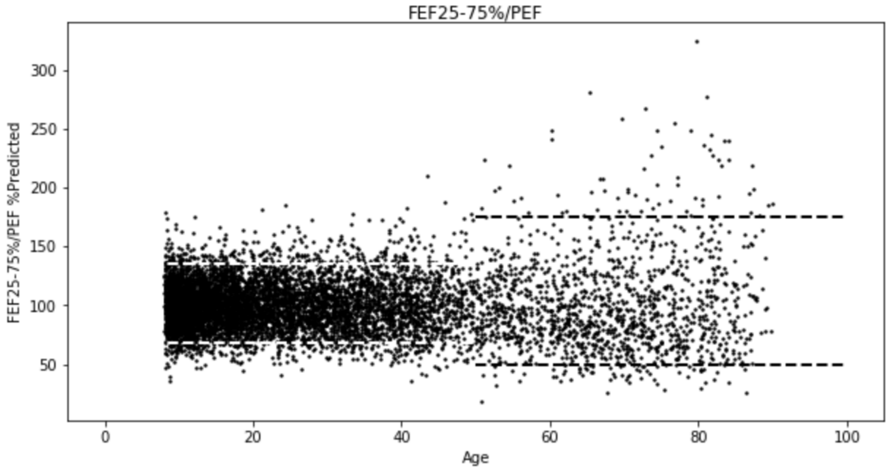
FEF25-75%/PEF
FEF25-75%/PEF %Predicted
300
250
200
150
100
50
0
20
40
60
80
100
Age

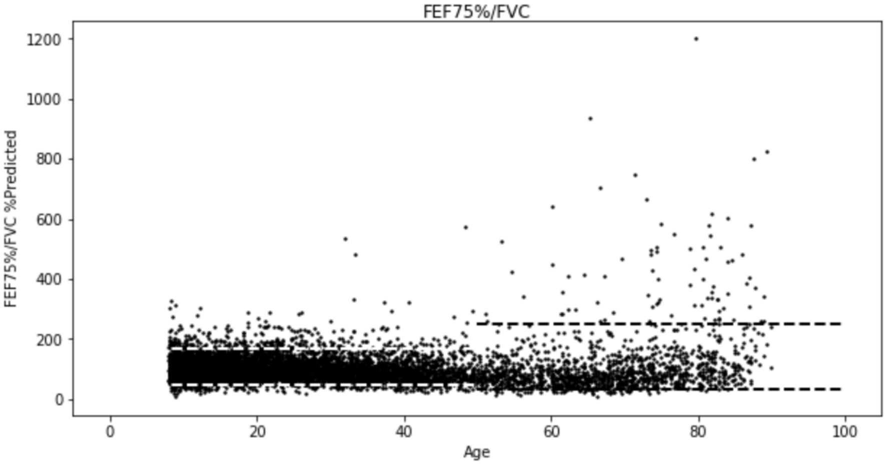
FEF75%/FVC
FEF75%/FVC %Predicted
1200
1000
800
600
400
200
0
0
20
40
60
80
100
Age

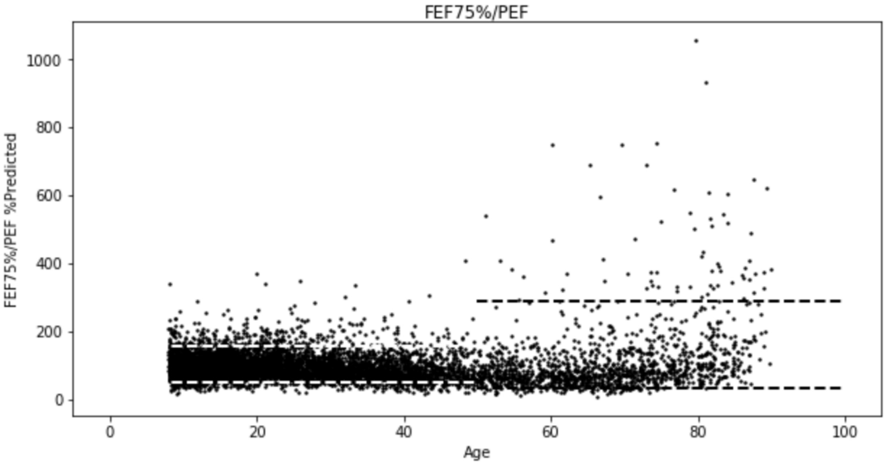
FEF75%/PEF
FEF75%/PEF %Predicted
1000
800
600
400
200
0
0
20
40
60
80
100
Age

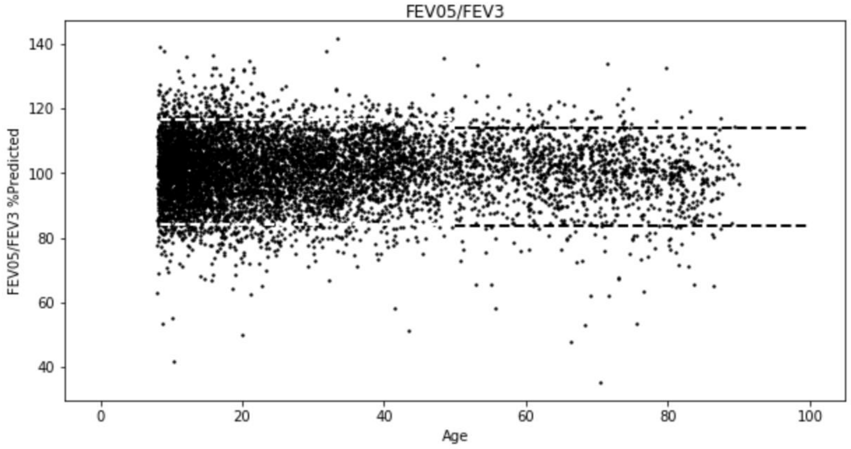
FEV05/FEV3
FEV05/FEV3 %Predicted
Age
140
120
100
80
60
40
0
20
40
60
80
100

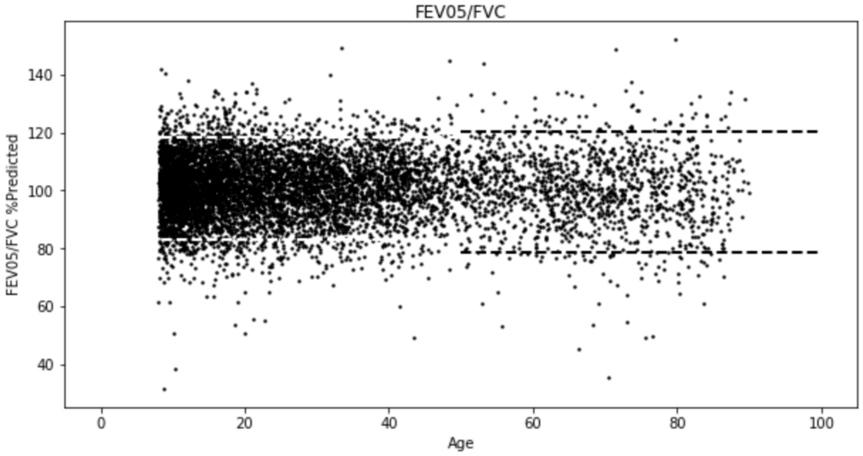
FEV05/FVC
FEV05/FVC %Predicted
Age
140
120
100
80
60
40
0
20
40
60
80
100

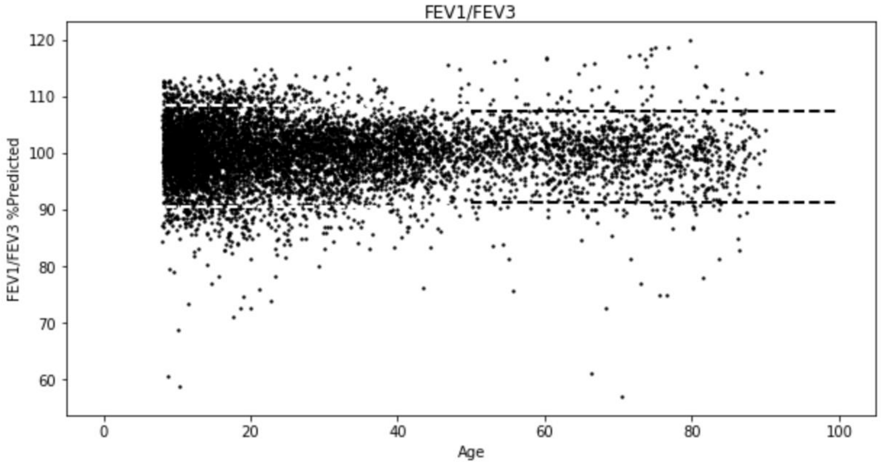
FEV1/FEV3
FEV1/FEV3 %Predicted
Age
120
110
100
90
80
70
60
0
20
40
60
80
100

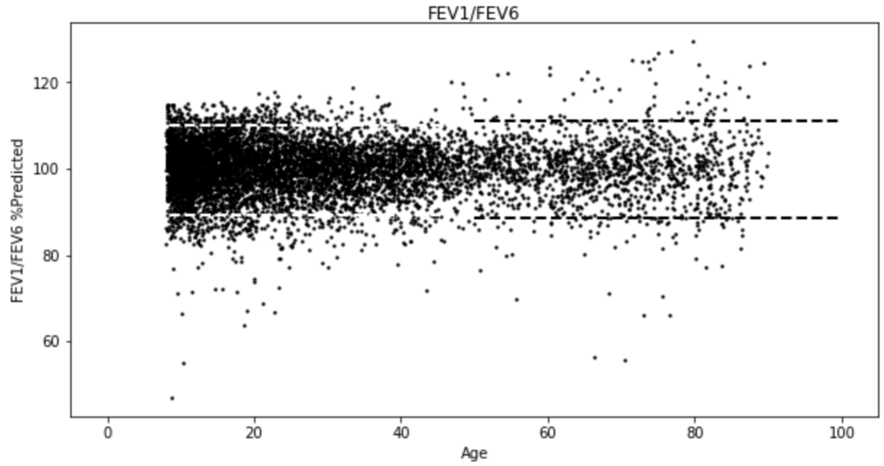
FEV1/FEV6
FEV1/FEV6 %Predicted
Age
120
100
80
60
0
20
40
60
80
100

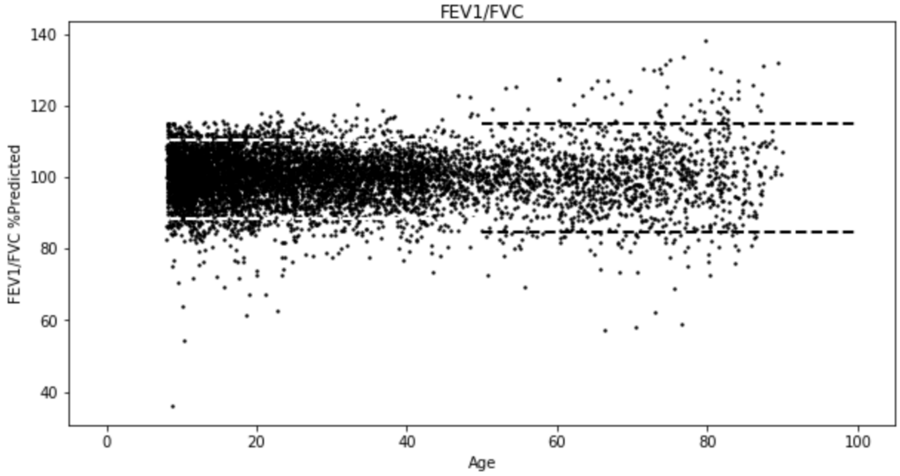
FEV1/FVC
FEV1/FVC %Predicted
Age
140
120
100
80
60
40
0
20
40
60
80
100

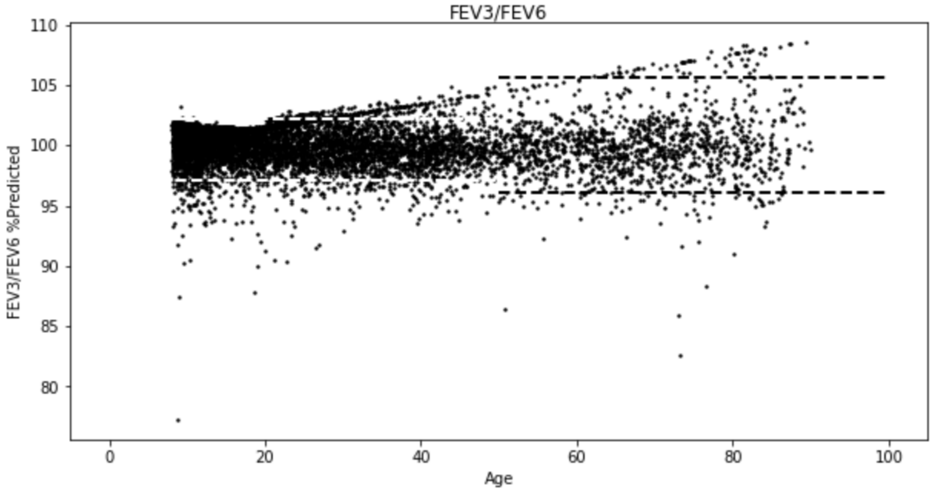
FEV3/FEV6
FEV3/FEV6 %Predicted
Age
110
105
100
95
90
85
80
0
20
40
60
80
100

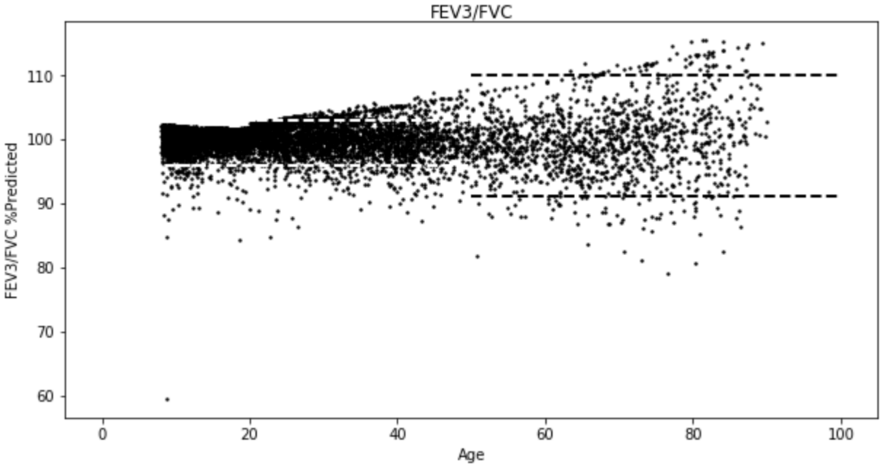
FEV3/FVC
FEV3/FVC %Predicted
110
100
90
80
70
60
0
20
40
60
80
100
Age

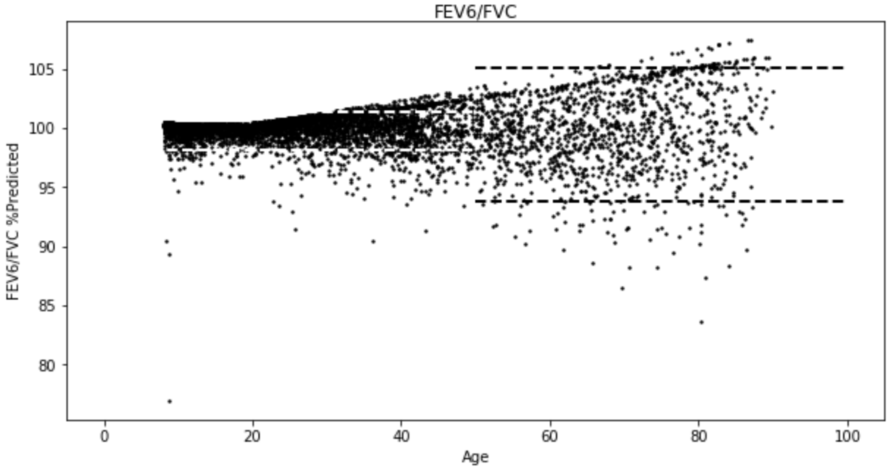
FEV6/FVC
FEV6/FVC %Predicted
105
100
95
90
85
80
0
20
40
60
80
100
Age

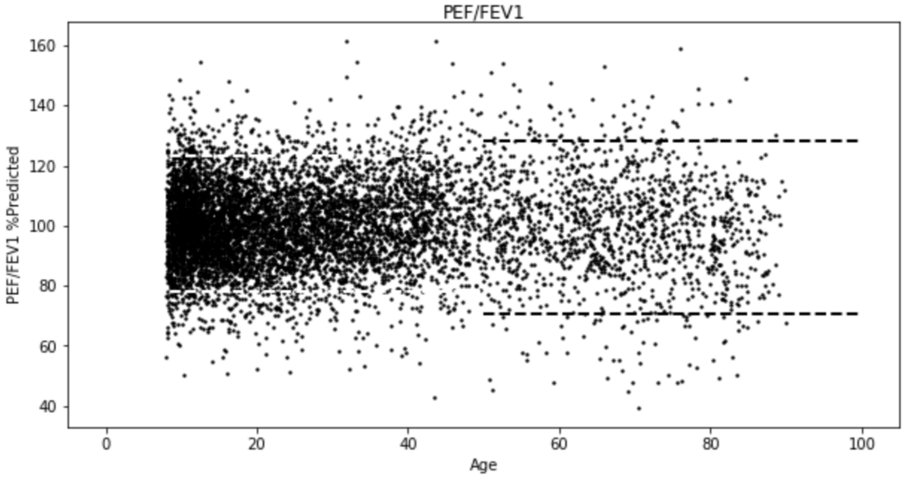
PEF/FEV1
PEF/FEV1 %Predicted
160
140
120
100
80
60
40
0
20
40
60
80
100
Age

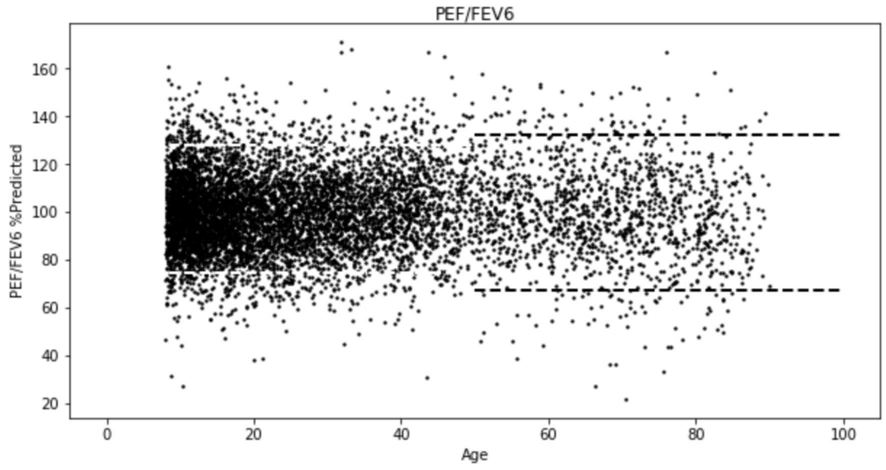

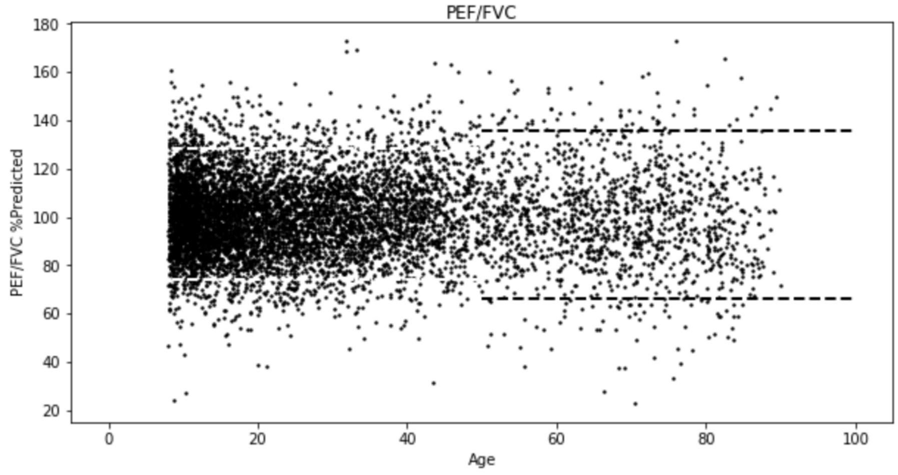

Appendix Fig. (1). Scatterplot (separate plot for each parameter) of percent of best predicted values by age, including lines for lower and upper limits of normal for age < 50 and for age ≥ 50.

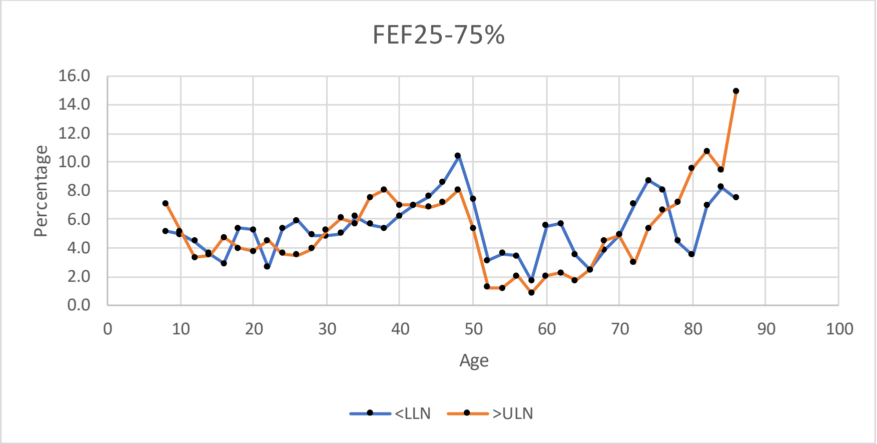

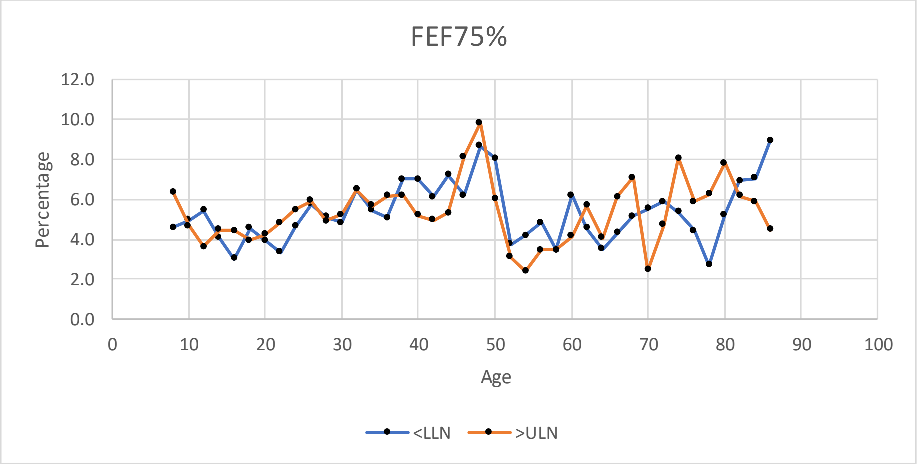
FEF75%
Percentage
12.0
10.0
8.0
6.0
4.0
2.0
0.0
0
10
20
30
40
50
60
70
80
90
100
Age
<LLN
>ULN

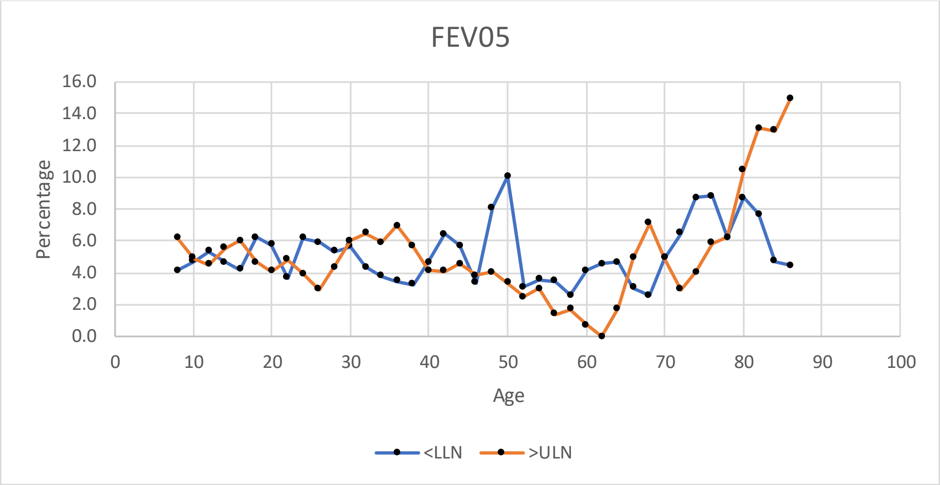
FEV05
Percentage
16.0
14.0
12.0
10.0
8.0
6.0
4.0
2.0
0.0
0
10
20
30
40
50
60
70
80
90
100
Age
<LLN
>ULN

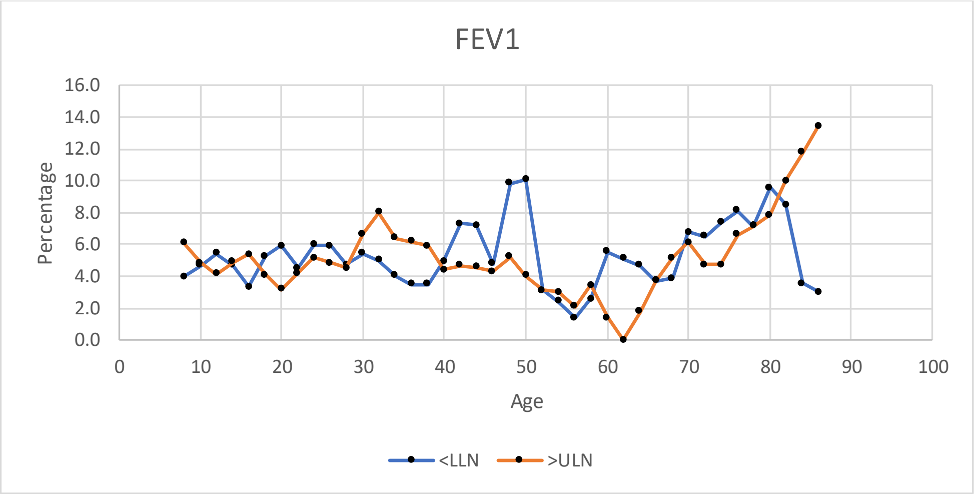
FEV1
Percentage
16.0
14.0
12.0
10.0
8.0
6.0
4.0
2.0
0.0
0
10
20
30
40
50
60
70
80
90
100
Age
<LLN
>ULN

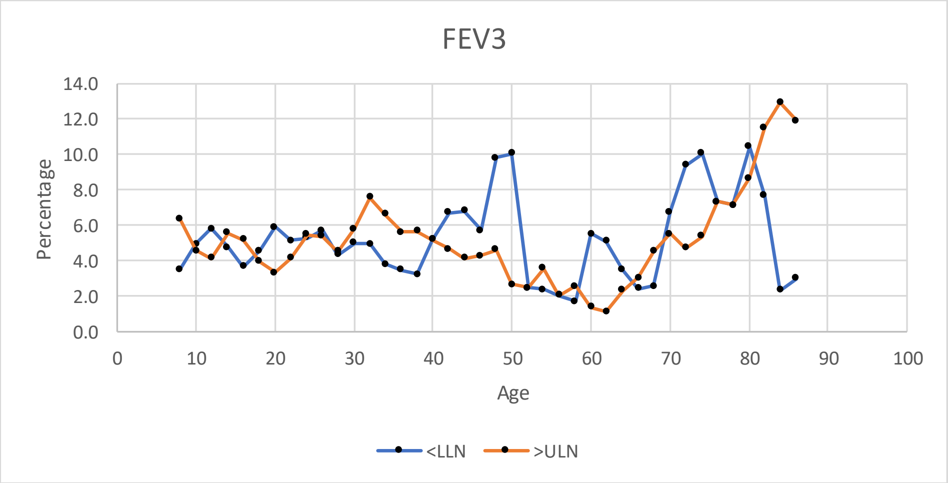
FEV3
Percentage
Age
<LLN
>ULN

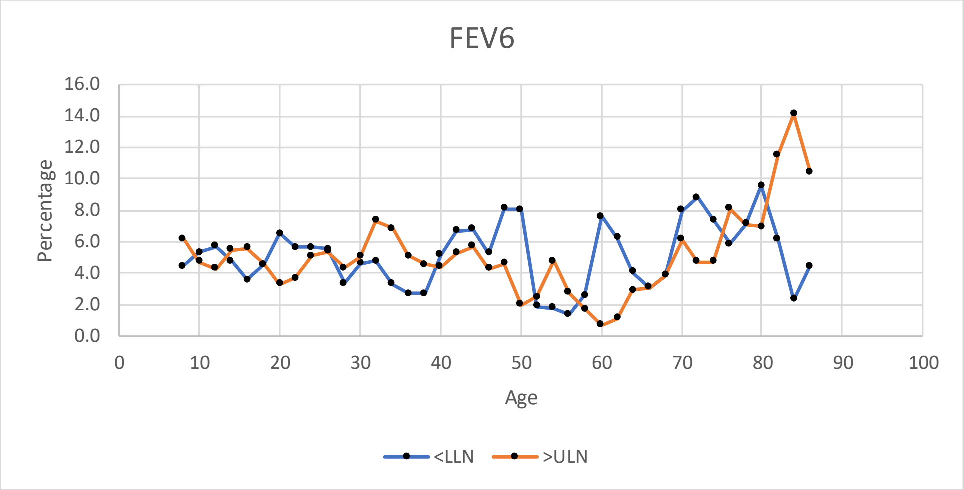
FEV6
Percentage
Age
<LLN
>ULN

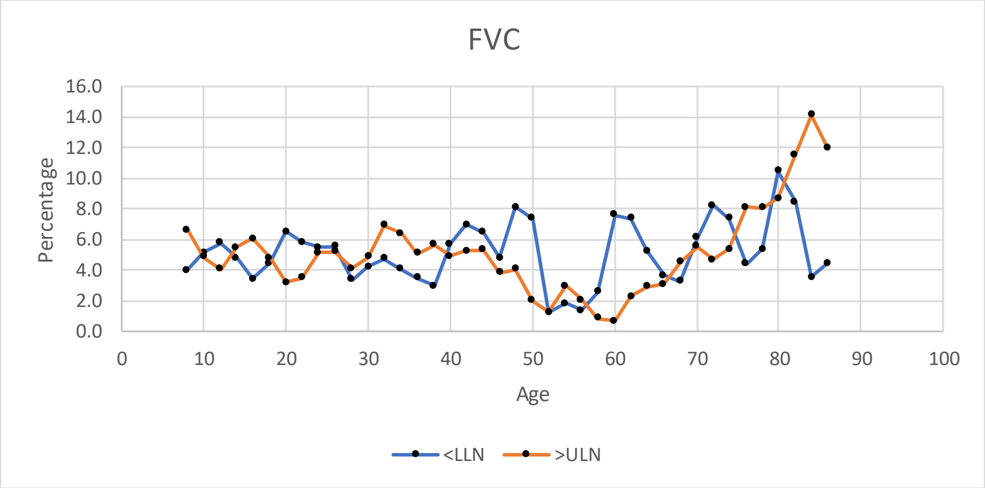
FVC
Percentage
Age
<LLN
>ULN

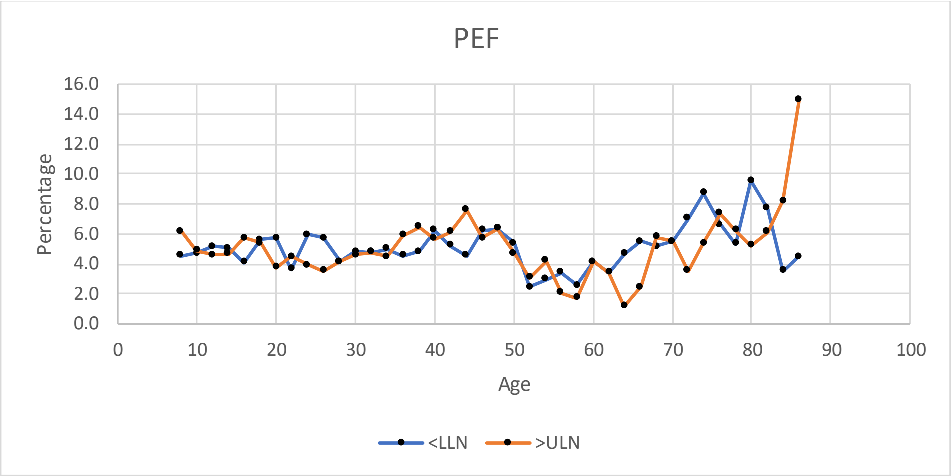
PEF
16.0
14.0
12.0
10.0
8.0
6.0
4.0
2.0
0.0
Percentage
0
10
20
30
40
50
60
70
80
90
100
Age
<LLN
>ULN

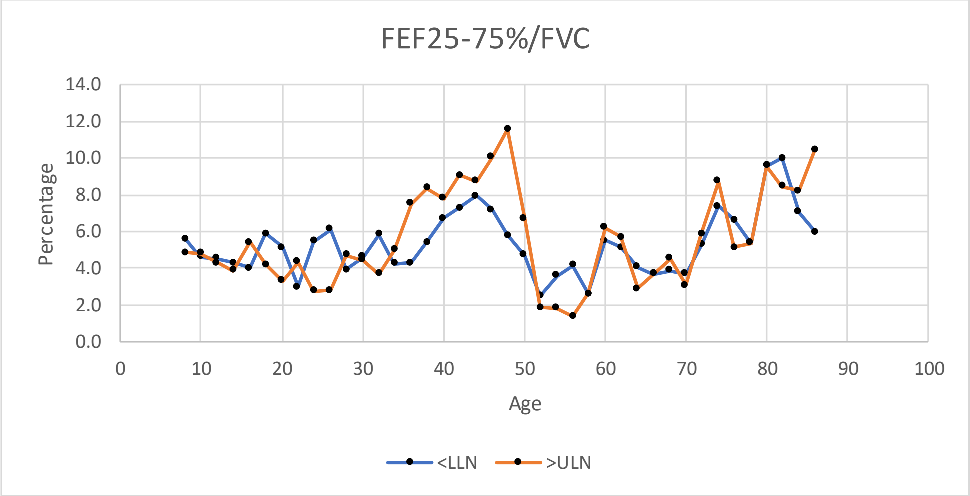
FEF25-75%/FVC
14.0
12.0
10.0
8.0
6.0
4.0
2.0
0.0
Percentage
0
10
20
30
40
50
60
70
80
90
100
Age
<LLN
>ULN

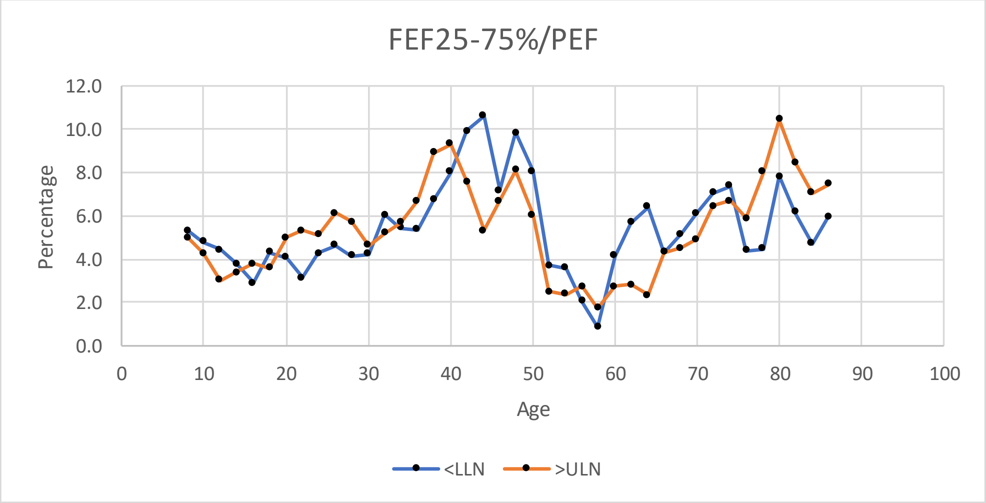
FEF25-75%/PEF
12.0
10.0
8.0
6.0
4.0
2.0
0.0
Percentage
0
10
20
30
40
50
60
70
80
90
100
Age
<LLN
>ULN

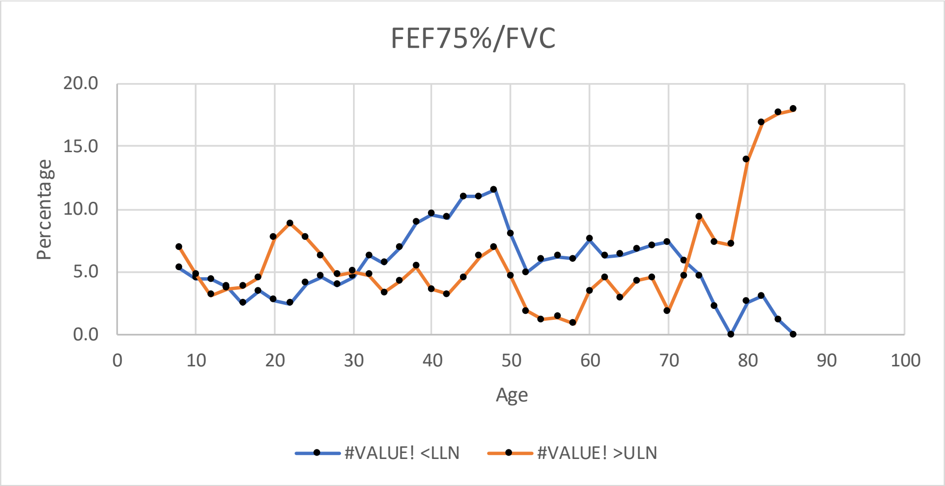
FEF75%/FVC
20.0
15.0
10.0
5.0
0.0
Percentage
0
10
20
30
40
50
60
70
80
90
100
Age
#VALUE! <LLN
#VALUE! >ULN

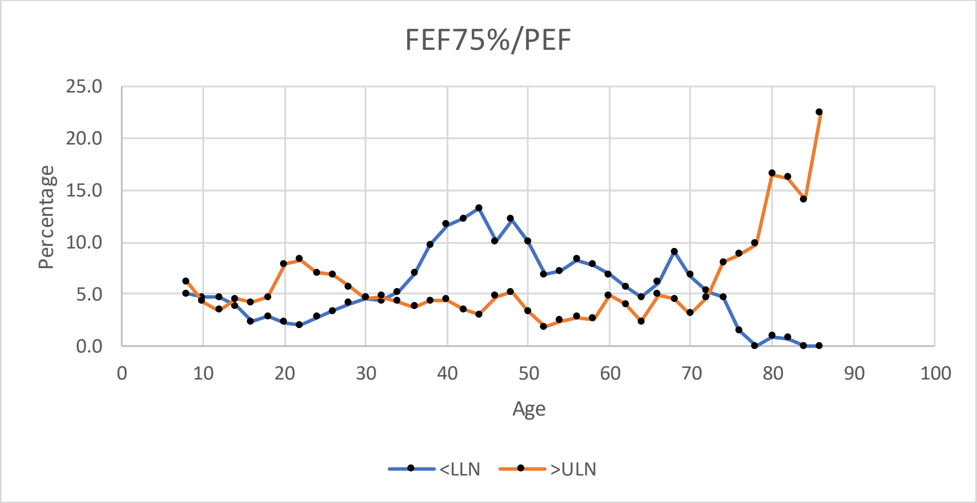
FEF75%/PEF
25.0
20.0
15.0
10.0
5.0
0.0
Percentage
0
10
20
30
40
50
60
70
80
90
100
Age
<LLN
>ULN

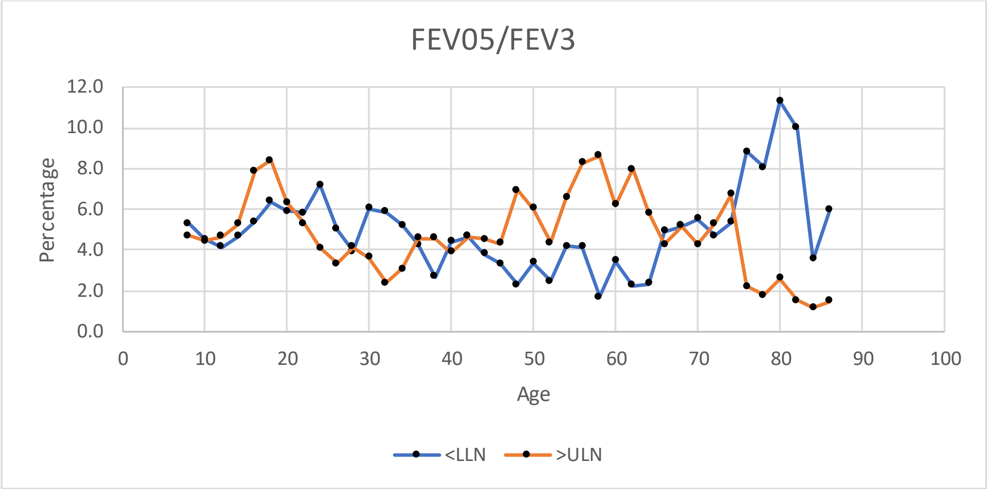
FEV05/FEV3
12.0
10.0
8.0
6.0
4.0
2.0
0.0
Percentage
0
10
20
30
40
50
60
70
80
90
100
Age
<LLN
>ULN

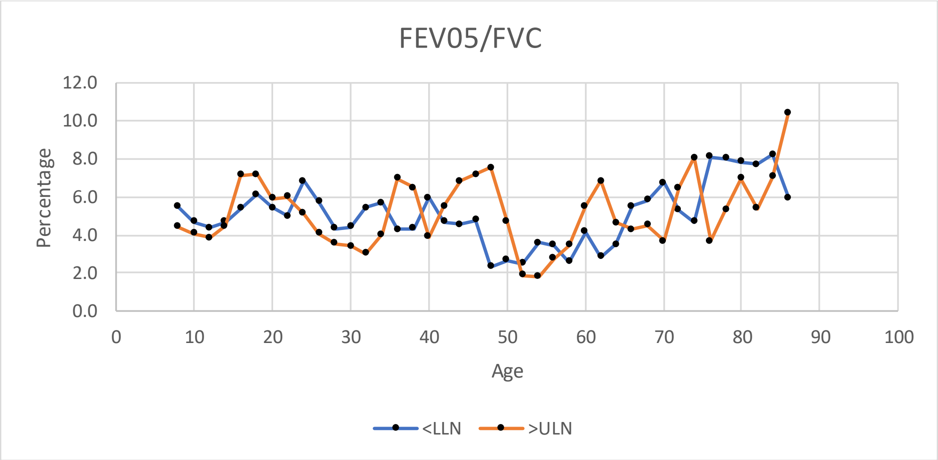
FEV05/FVC
Percentage
12.0
10.0
8.0
6.0
4.0
2.0
0.0
0
10
20
30
40
50
60
70
80
90
100
Age
<LLN
>ULN

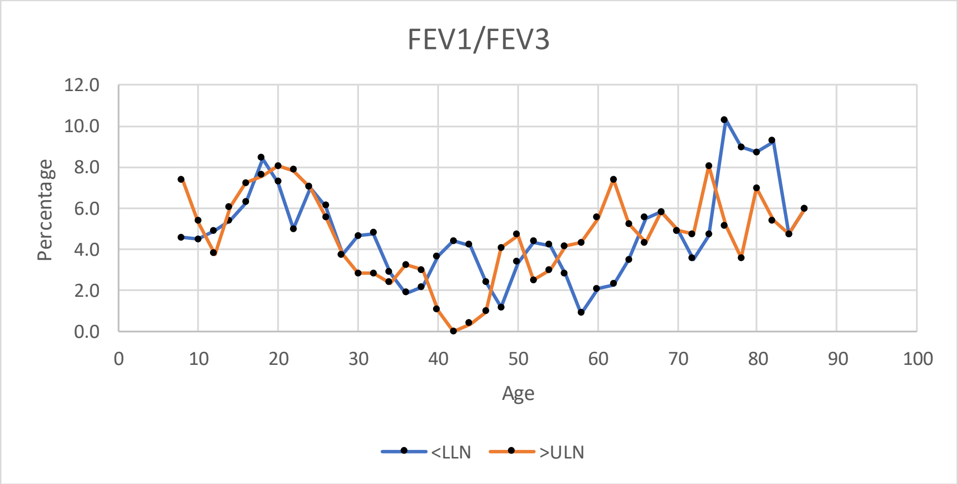
FEV1/FEV3
Percentage
12.0
10.0
8.0
6.0
4.0
2.0
0.0
0
10
20
30
40
50
60
70
80
90
100
Age
<LLN
>ULN

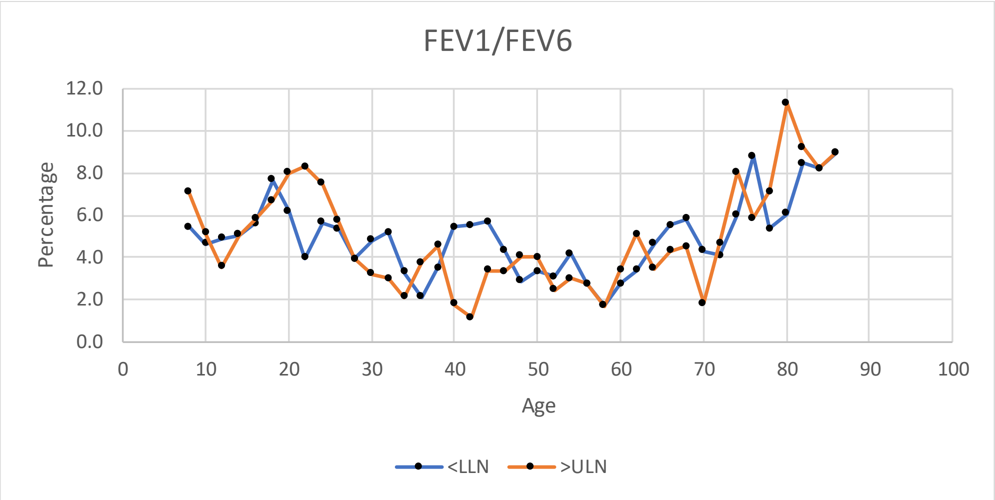
FEV1/FEV6
Percentage
12.0
10.0
8.0
6.0
4.0
2.0
0.0
0
10
20
30
40
50
60
70
80
90
100
Age
<LLN
>ULN

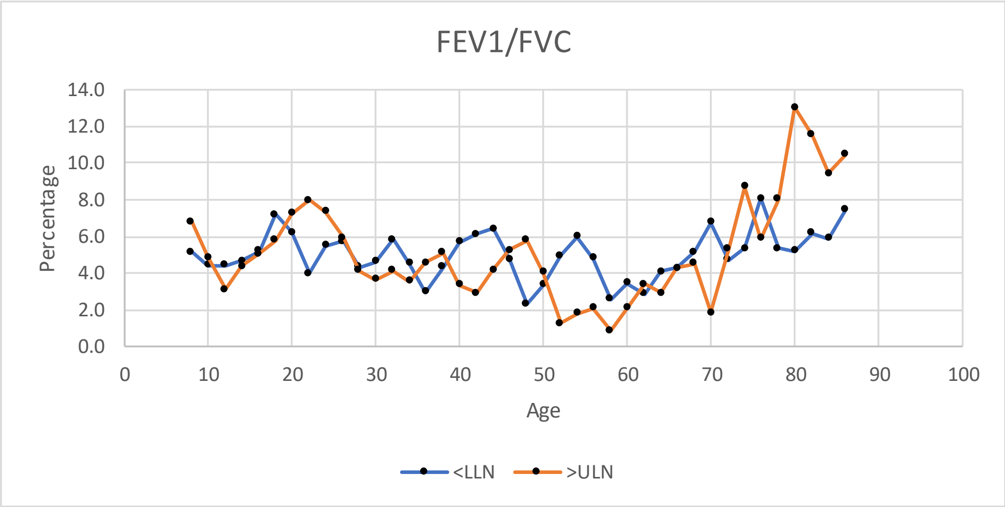
FEV1/FVC
Percentage
Age
<LLN
>ULN

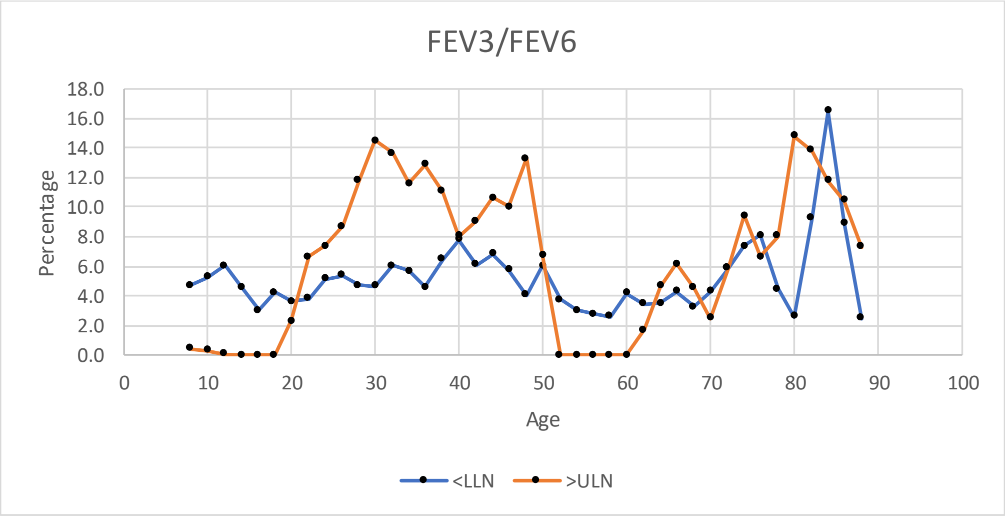
FEV3/FEV6
Percentage
Age
<LLN
>ULN

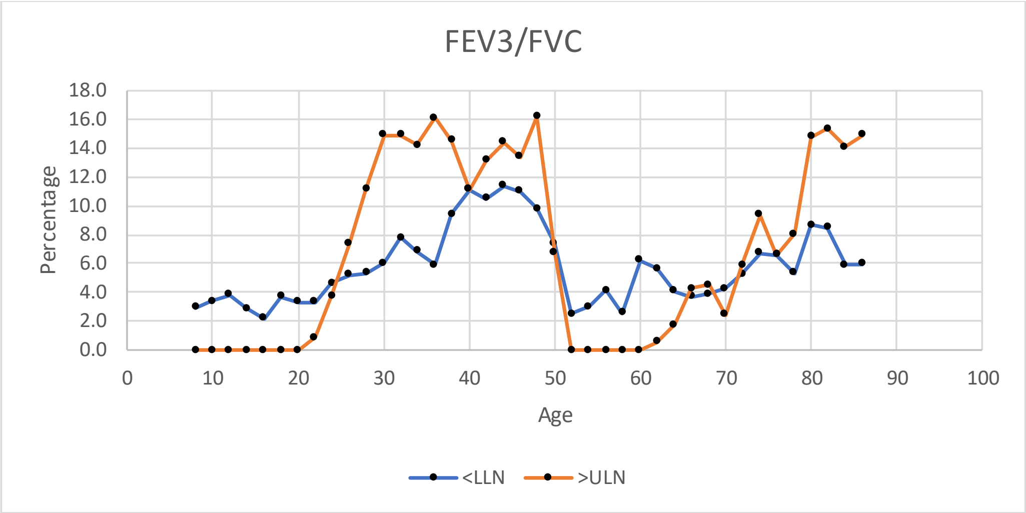
FEV3/FVC
Percentage
Age
<LLN
>ULN

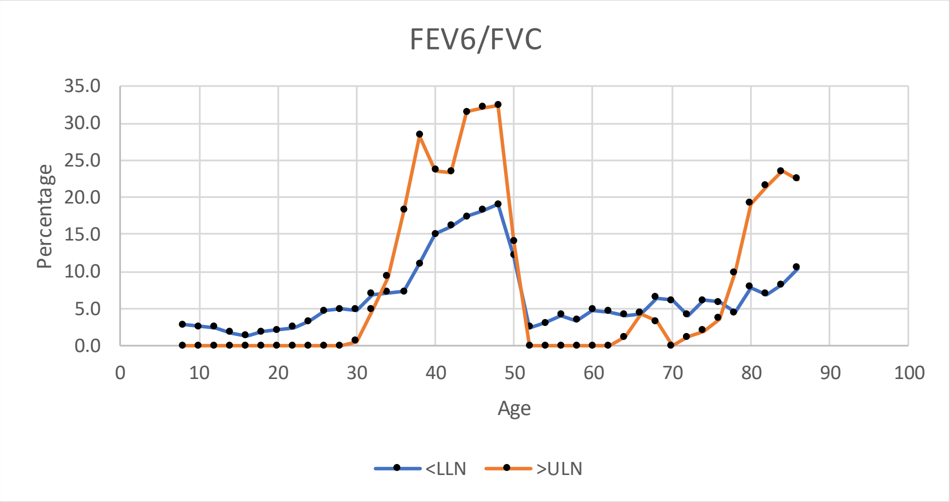
FEV6/FVC
35.0
30.0
25.0
20.0
15.0
10.0
5.0
0.0
Percentage
0
10
20
30
40
50
60
70
80
90
100
Age
<LLN
>ULN

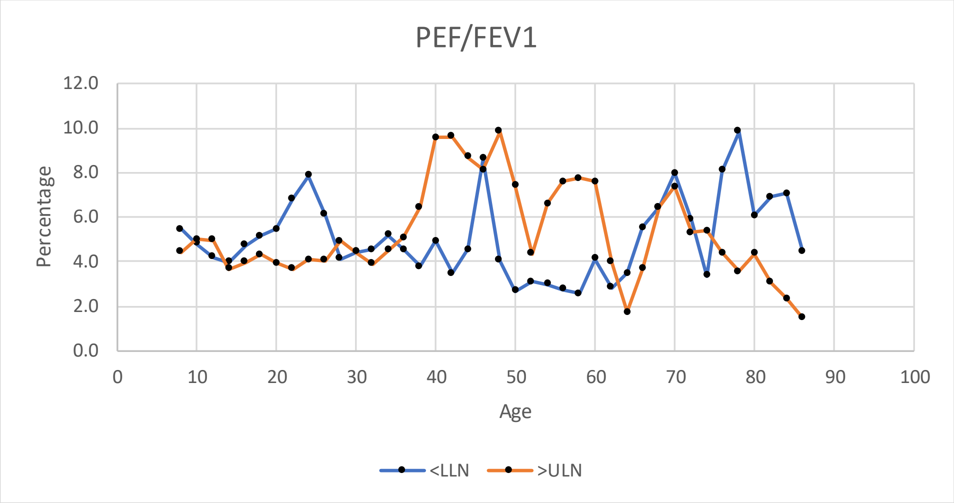
PEF/FEV1
12.0
10.0
8.0
6.0
4.0
2.0
0.0
Percentage
0
10
20
30
40
50
60
70
80
90
100
Age
<LLN
>ULN

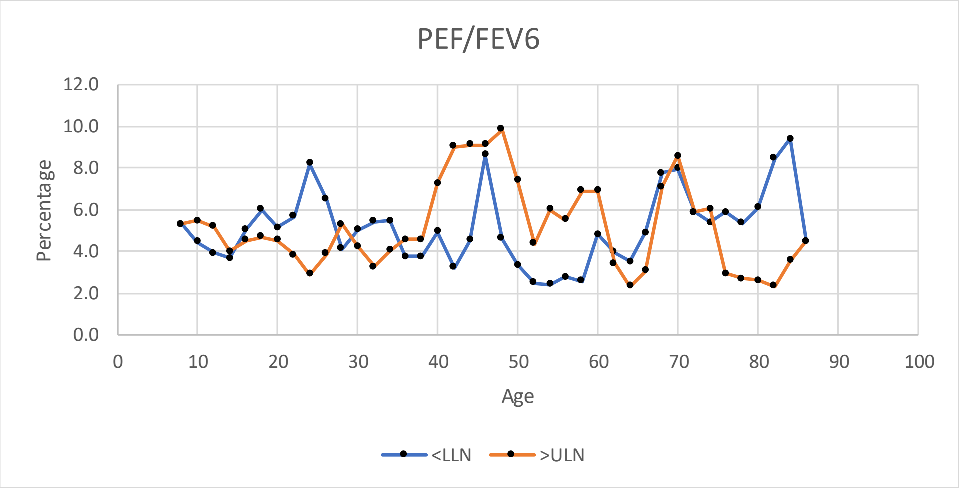
PEF/FEV6
12.0
10.0
8.0
6.0
4.0
2.0
0.0
Percentage
0
10
20
30
40
50
60
70
80
90
100
Age
<LLN
>ULN

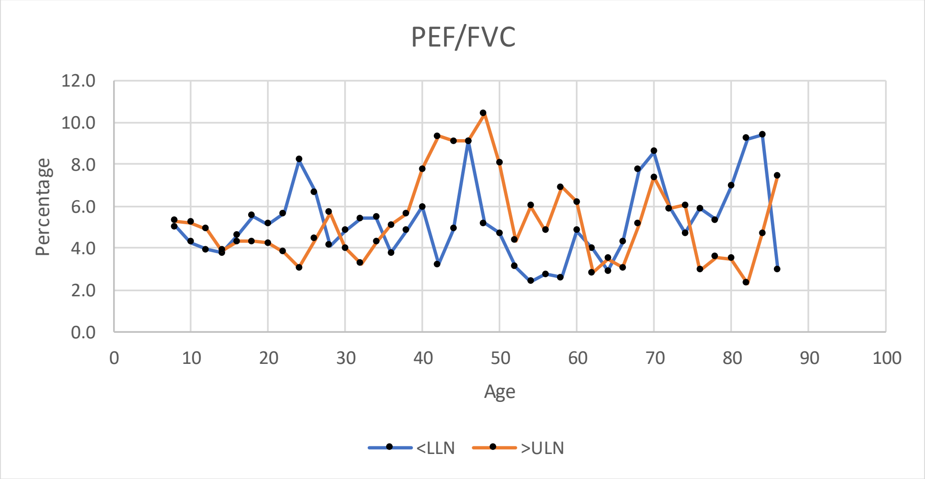

Appendix Fig. (2). Percentage of subjects below predicted lower limit of normal (LLN) or above predicted upper limit of normal (ULN) by age (those ± 2 years, overlapping 4-year intervals) for all parameters.

© 2023 Clark Johnson and Gardner Johnson

This is an open access article distributed under the terms of the Creative Commons Attribution 4.0 International Public License (CC-BY 4.0), a copy of which is available at: https://creativecommons.org/licenses/by/4.0/legalcode. This license permits unrestricted use, distribution, and reproduction in any medium, provided the original author and source are credited.
